# Single-cell profiling of vascular endothelial cells reveals progressive organ-specific vulnerabilities during obesity

**DOI:** 10.1038/s42255-022-00674-x

**Published:** 2022-11-18

**Authors:** Olga Bondareva, Jesús Rafael Rodríguez-Aguilera, Fabiana Oliveira, Longsheng Liao, Alina Rose, Anubhuti Gupta, Kunal Singh, Florian Geier, Jenny Schuster, Jes-Niels Boeckel, Joerg M. Buescher, Shrey Kohli, Nora Klöting, Berend Isermann, Matthias Blüher, Bilal N. Sheikh

**Affiliations:** 1 Helmholtz Institute for Metabolic, Obesity and Vascular Research (HI-MAG) of the Helmholtz Center Munich, Leipzig, Germany; 2 Medical Faculty, University of Leipzig, Leipzig, Germany; 3 Institute of Laboratory Medicine, Clinical Chemistry, and Molecular Diagnostics, University Hospital Leipzig, Leipzig, Germany; 4 Klinik und Poliklinik für Kardiologie, Universitätsklinikum Leipzig, University of Leipzig, Leipzig, Germany; 5 Max Planck Institute for Immunobiology and Epigenetics, Freiburg im Breisgau, Germany; 6 Medical Department III—Endocrinology, Nephrology, Rheumatology, University of Leipzig, Leipzig, Germany

**Keywords:** Obesity, Vascular diseases

## Abstract

Obesity promotes diverse pathologies, including atherosclerosis and dementia, which frequently involve vascular defects and endothelial cell (EC) dysfunction. Each organ has distinct EC subtypes, but whether ECs are differentially affected by obesity is unknown. Here we use single-cell RNA sequencing to analyze transcriptomes of ~375,000 ECs from seven organs in male mice at progressive stages of obesity to identify organ-specific vulnerabilities. We find that obesity deregulates gene expression networks, including lipid handling, metabolic pathways and AP1 transcription factor and inflammatory signaling, in an organ- and EC-subtype-specific manner. The transcriptomic aberrations worsen with sustained obesity and are only partially mitigated by dietary intervention and weight loss. For example, dietary intervention substantially attenuates dysregulation of liver, but not kidney, EC transcriptomes. Through integration with human genome-wide association study data, we further identify a subset of vascular disease risk genes that are induced by obesity. Our work catalogs the impact of obesity on the endothelium, constitutes a useful resource and reveals leads for investigation as potential therapeutic targets.

## Abstract (web summary)

Bondareva and Rodríguez-Aguilera et al. use scRNA-seq to analyze transcriptomes of 375,000 endothelial cells from seven organs in male mice at various stages of obesity to identify organ-specific vulnerabilities.

## Main

Obesity is rapidly increasing worldwide due to changing diets and lifestyles, with 650 million adults classified as obese^1,2^. Obesity promotes the development of numerous acute and chronic diseases, including atherosclerosis, heart failure, neurodegeneration, stroke, pulmonary hypertension, renal vascular disease, microvascular dysfunction and a range of hepatic vascular complications^3–7^. All of these disorders are associated with vascular defects, suggesting that vascular dysfunction in obesity represents a shared mechanism of disease.

Blood vessels are lined by endothelial cells (ECs), which control the transport of nutrients, metabolites, oxygen and carbon dioxide between the blood and organs^8^. Obesity can cause global EC dysfunction, characterized by reduced nitric oxide bioavailability and increased oxidative damage^9,10^. However, ECs in different organs are functionally and molecularly distinct^11,12^. Additionally, dehydration differentially affects the transcriptomes of kidney ECs found in the glomerular, medullary and cortical compartments^13^. Overall, these observations suggest that the same physiological trigger can have unique impacts on different EC subtypes. Nevertheless, how obesity affects the distinct molecular networks in ECs from different organs is unclear. This knowledge is critical to understand the mechanisms of vascular dysfunction in metabolic disease.

In addition, to what extent the trajectory of obesity-induced changes in endothelium can be mitigated by lifestyle interventions is unclear. One of the best clinical strategies for reducing the risk of comorbidities in individuals with obesity is weight loss^14^. However, despite severe interventions, such as bariatric surgery and associated weight loss due to dietary restriction, the improvement in health and long-term life expectancy remains limited^15^. From a clinical perspective, it is critical to identify the molecular networks that are resistant to improved metabolic health, as they could serve as therapeutic targets aimed at ameliorating high-risk profiles associated with metabolic disease.

To address these outstanding questions, we used single-cell RNA sequencing (scRNA-seq) to systematically map the impact of obesity on the endothelium in mice. We reveal unique organ- and EC-subtype-specific molecular changes driven by obesity. By switching obese animals onto a normal chow diet, we uncovered distinct obesity-driven transcriptome changes in ECs that are responsive or resistant to weight loss. In addition, we analyzed human genome-wide association studies (GWASs) and identified high-risk disease genes that become dysregulated in ECs with sustained obesity. This study provides an extensive resource of obesity-driven changes in the endothelium and identifies genes that potentiate the risk of obesity-associated disorders. The data generated in this study are available through an interactive website at https://obesity-ecatlas.helmholtz-muenchen.de.

## Results

### Obesity induces organ-specific changes in ECs

**Fig. 1:**
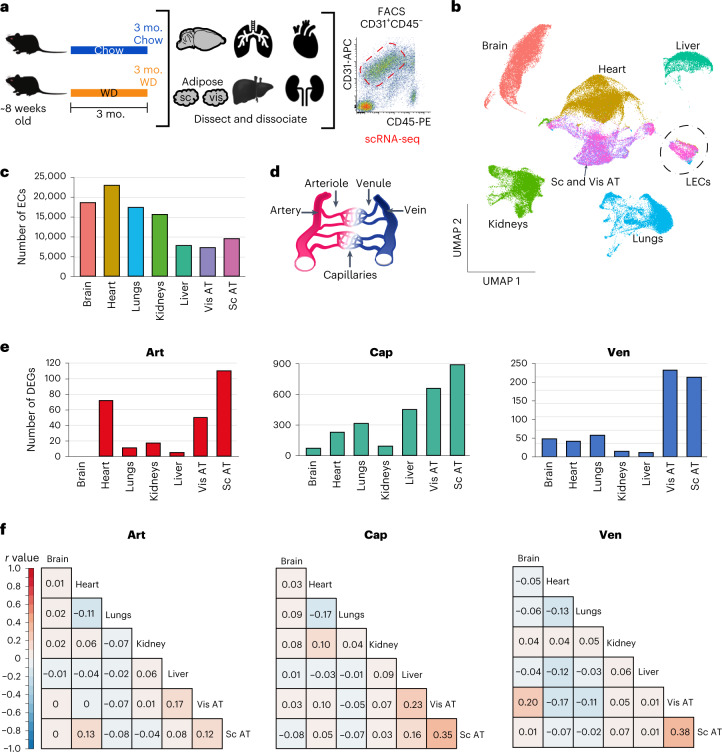
Obesity induces organ-specific changes in ECs. **a**, Experimental design; *n* = 3 animals per group. The FACS plot shows exemplary gating for CD31^+^CD45^low^ cells; sc, subcutaneous; vis, visceral; mo., months. **b**, Uniform manifold approximation and projection (UMAP) clustering of ECs from seven organs of mice on a WD or chow diet after filtering. Colors correspond to the organ from which the ECs were derived. Each dot represents a single EC; *n* = 3 animals per diet. **c**, Number of ECs analyzed from each organ after filtering out low-quality cells and non-ECs. **d**, Schematics of major vessel types. **e**, Number of DEGs (adjusted *P* value of <0.05 and | log (fold change (FC)) | > 0.1) in ECs from the different organs of obese versus control mice in (1) art, (2) cap and (3) ven. **f**, Correlation of gene expression changes in obese versus control conditions across art, cap and ven ECs. Genes showing a | log (FC) | > 0.1 in any tissue for the indicated EC population were used to collate the list of genes used for these analyses. A Pearson *r* value for each comparison is provided. Adjusted *P* value indicates adjustments for multiple comparisons using the Benjamini–Hochberg method (**e**). Source data

**Extended Data Fig. 1:**
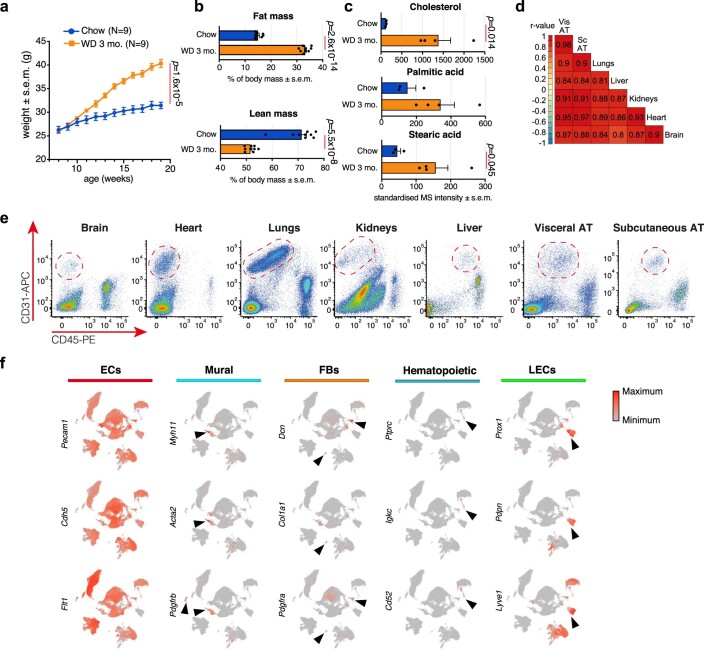
Isolation and bioinformatical filtering of ECs. (**a**) Mouse body weight over 3 months of WD. N = 9 animals per group. (**b**) Percentage of lean versus fat mass in animals maintained on WD and chow diets for 3 months. N = 9 animals per group. (**c**) Metabolomics (GC-MS) data showing serum cholesterol, palmitate and stearic acid levels in obese versus control animals. N = 3 animals on chow diet, 4 on WD. (**d**) Correlation matrix comparing the overall EC transcriptome from the 7 indicated organs, with WD and chow groups combined. Pearson’s r-value for each comparison is provided. (**e**) Representative FACS plots showing sorting strategy for ECs at the 3-month timepoint. Single cells were selected based on forward and side scatter, dead cells removed based on propidium iodide staining, and enriched populations of ECs isolated based on high CD31 (PECAM1) and low CD45 levels. (**f**) UMAPs showing the presence of vascular EC, mural, fibroblast (FB), hematopoietic and lymphatic EC (LEC) markers across all CD31^+^ CD45^low^ cell analyzed by scRNA-seq. The arrows indicate the positive population in each category. Data are presented as mean ± SEM and were analyzed using a two-sided Student’s t-test (**a-c**). Source data

To determine the impact of obesity on vascular ECs, we fed 8-week-old mice a Western diet (WD) or control chow diet for 3 months (Fig. 1a). WD led to a significant increase in body weight and percent body fat, while metabolomics analysis revealed an increase in serum cholesterol and stearic acid (Extended Data Fig. 1a–c), confirming the expected obesity phenotypes. We used fluorescence-activated cell sorting (FACS) to isolate ECs (CD31^+^CD45^low^) from seven major organs (brain, heart, lungs, kidneys, liver, visceral adipose tissue (AT) and subcutaneous AT) and performed scRNA-seq (Fig. 1a and Extended Data Fig. 1d,e).

ECs were discerned from contaminating cell types based on the expression of well-established markers^16^. Cells expressing *Pecam1* (*Cd31*), *Cdh5* and *Flt1* were classified as ECs, whereas mural cells (positive for *Myh11*, *Acta2* and *Pdgfrb*), fibroblasts (*Dcn*, *Col1a1* and *Pdgfra*) and immune cells (*Ptprc*, *Igkc* and *Cd52*) were removed from further analyses (Extended Data Fig. 1f). Additionally, cells with low counts (<500 unique genes) or high mitochondrial transcripts (>20% of all transcripts) were filtered out (Supplementary Table 1). After filtering, we analyzed between 7,000 and 23,000 ECs for each organ (Fig. 1b,c). We collated an initial database of 99,739 ECs from obese and control mice, which we used for our first set of analyses.

**Extended Data Fig. 2:**
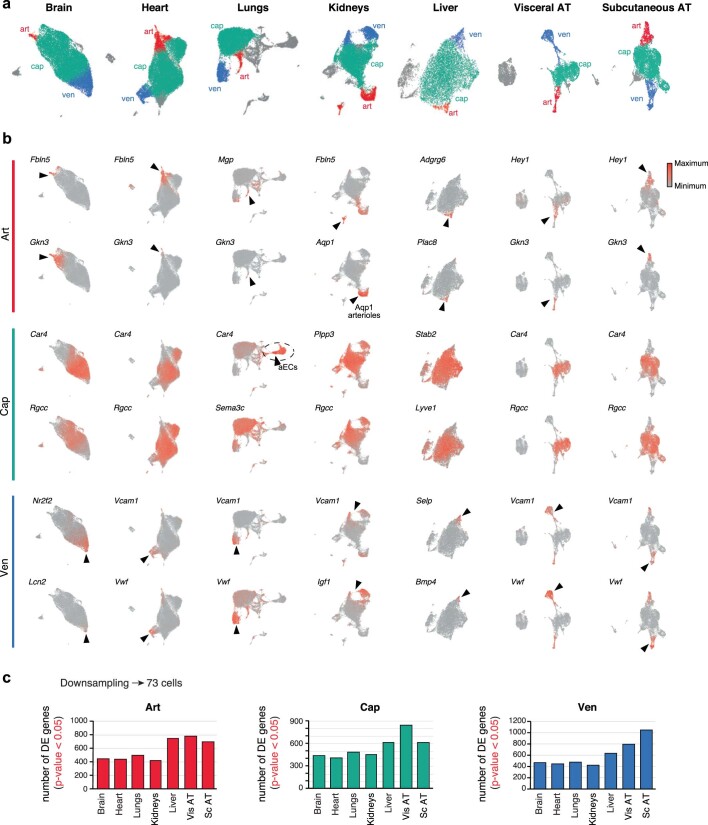
Identification of major EC subtypes across organs. (**a**) Assignment of arterial + arteriole (art), capillary (cap) and veins + venule (ven) identities to ECs in each organs. The identities were defined based on markers presented in panel (**b**). Cells indicated in gray are specialized ECs that don’t broadly fit into the art, cap and ven EC categories. (**b**) UMAPs showing representative markers of art, cap and ven ECs for each organ. All markers, including the representatives shown here, were derived from published scRNA-seq datasets^11,13,16–18^. Arrows mark the respective positive cell populations. (**c**) Number of differentially expressed genes (DEGs) in the art, cap and ven ECs after down-sampling. Down-sampling to 73 ECs was done to ensure equal statistical power in each group. As down-sampling also resulted in lower statistical power, a lenient cut-off of *p* < 0.05 (rather than Benjamini-Hochberg adjusted *p*-value < 0.05) was used. Source data

Although ECs isolated from different organs had similar transcriptional profiles (*r* > 0.8; Extended Data Fig. 1d), they segregated into distinct groups based on unbiased clustering (Fig. 1b), consistent with the unique physiological functions of ECs in different organs. We classified vascular ECs from each organ into three broad subgroups based on established markers^11,16,17^, including (1) arteries and arterioles (‘art’), (2) capillaries (‘cap’) and (3) veins and venules (‘ven’; Fig. 1d and Extended Data Fig. 2a,b). We then calculated the number of differentially expressed genes (DEGs) in obese versus control mice. Notably, AT ECs showed the highest number of DEGs, particularly in cap ECs (Fig. 1e), followed by liver cap ECs. As statistical testing is impacted by the number of ECs in each cluster, we downsampled to 73 cells, the lowest number of ECs in any cluster, to equalize statistical power and quantified DEGs. These analyses confirmed that AT and liver ECs were the most impacted by obesity (Extended Data Fig. 2c). Together, these data are consistent with the AT and liver acting as major hubs of lipid metabolism.

We next identified the top DEGs in ECs from each organ in obese animals. There was some correlation between the DEGs in ECs from visceral AT and subcutaneous AT (cap ECs *r* = 0.35 and ven ECs *r* = 0.38) and from visceral AT and liver (cap ECs *r* = 0.23; Fig. 1f). Overall, however, obesity-induced DEGs displayed low concordance between ECs from different organs, suggesting that obesity impacts the endothelium in an organ-specific manner. To determine how obesity uniquely impacts ECs in each organ, we performed unbiased clustering of ECs in each organ, assigned their identities based on known markers^11,13,16–18^ and investigated obesity-induced changes in EC clusters.

#### AT ECs

**Fig. 2:**
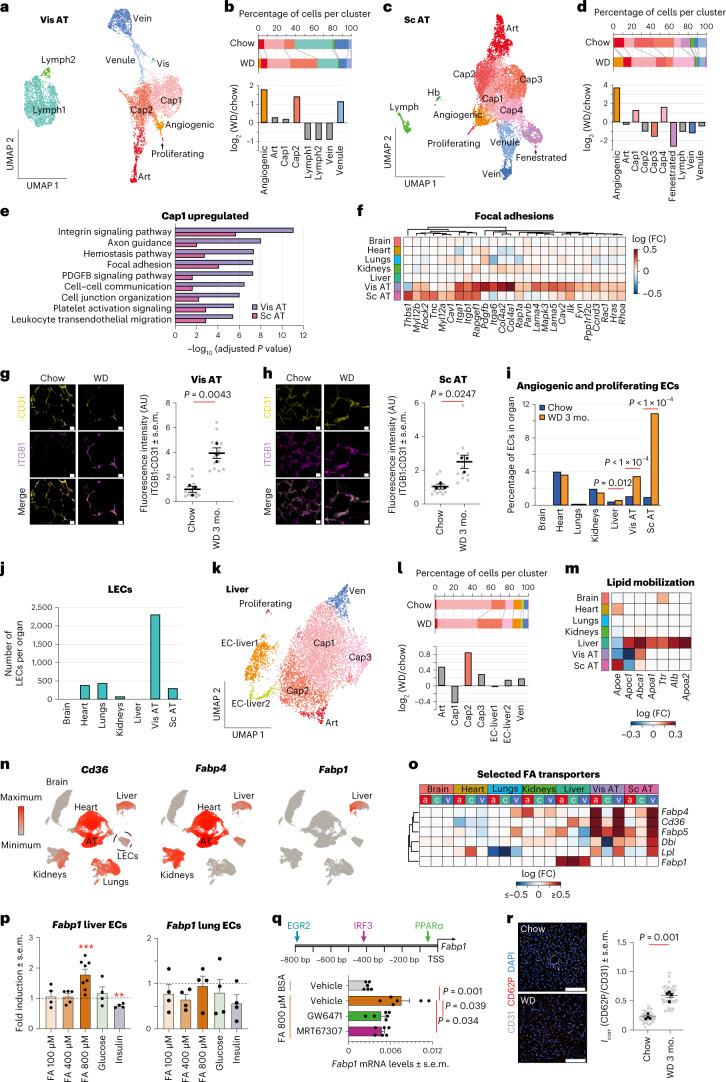
Obesity induces ECM remodeling, angiogenesis and lipid transporters in AT and liver ECs. **a**, UMAP clustering of visceral AT ECs. **b**, Shifts in EC populations in visceral AT. Populations showing a greater than twofold change in obesity are highlighted in color. **c**, UMAP clustering of subcutaneous AT ECs. **d**, Shifts in EC clusters in subcutaneous AT. Populations showing a greater than twofold change in obesity are highlighted in color. **e**, BioPlanet-annotated pathways upregulated in visceral and subcutaneous AT cap1 ECs in obesity. Significantly upregulated (adjusted *P* value of <0.05) genes were used for these analyses. **f**, Expression changes in focal adhesion-related genes in cap ECs in obese versus control animals. **g**,**h**, Immunostaining of integrin-β1 (ITGB1) and CD31 in visceral (**g**) and subcutaneous AT (**h**); *n* = 3 animals per group (black dots); *n* = 4 to 5 sections per animal (gray dots); scale bars, 20 µm; AU, arbitrary units. **i**, Quantification of proliferating and angiogenic ECs. Data were analyzed using a two-sided *χ*^2^ test. **j**, Number of LECs detected per organ. **k**, UMAP clustering of liver ECs. **l**, Shifts in EC liver populations. Populations showing a log_2_ (WD/chow) > 0.5 change are highlighted in color. **m**, Changes in select lipid mobilization genes in cap ECs of obese animals. **n**, UMAPs showing enrichment of fatty acid transporters. **o**, Changes in fatty acid (FA) transporters in art (a), cap (c) and ven (v) ECs in obesity. **p**, *Fabp1* mRNA expression in response to free fatty acids, glucose and insulin; *n* = 4–8 replicates per group. Treatments were compared against BSA-treated controls; **P = 0.003 and ***P = 0.0003. **q**, Predicted transcription factor binding sites in the *Fabp1* promoter (top) and impact of PPARα (GW6471) and TBK1/IKKε (MRT67307) inhibitors on fatty acid-driven *Fabp1* activation (bottom); *n* = 6 replicates per group; bp, base pairs; TSS, transcription start site. **r**, Colocalization of CD62P and CD31 in livers from obese versus control mice; *n* = 4 animals per group (black dots); *n* = 10 sections per animal (gray dots); scale bars, 100 µm. Data in **g**, **h**, **p**, **q** and **r** are presented as mean ± s.e.m. and were analyzed using a two-sided Student’s *t*-test. Expression data in **p** and **q** were standardized to *Gapdh* and *Rplp0*. The adjusted *P* value indicates adjustments for multiple comparisons using the Benjamini–Hochberg method (**e**). Source data

**Extended Data Fig. 3:**
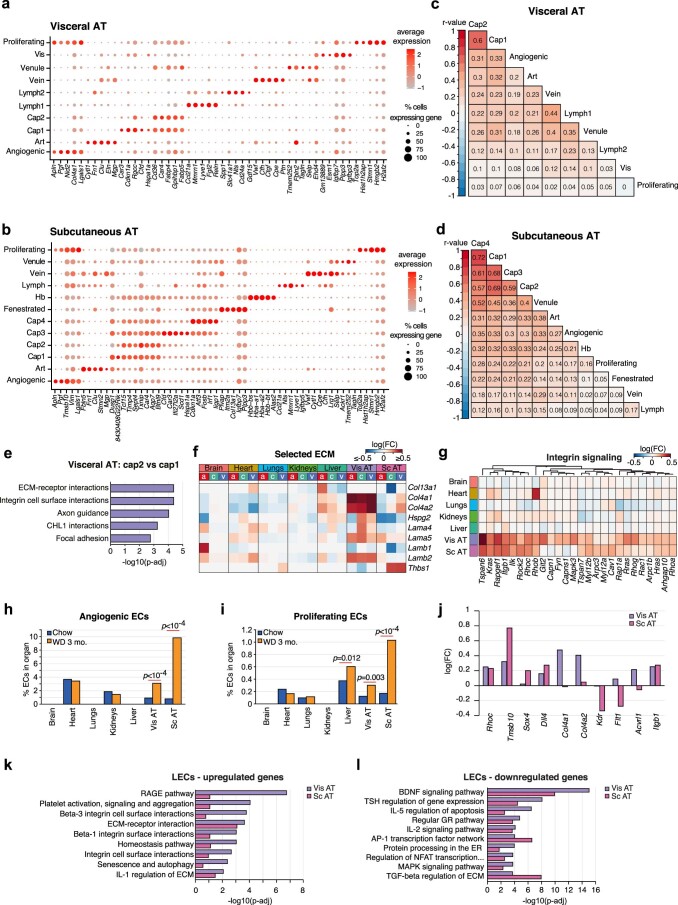
Obesity induces ECM genes in adipose tissue ECs. (**a-b**) Top 5 enriched genes in (**a**) visceral and (**b**) subcutaneous AT EC clusters. (**c-d**) Comparison of obesity-induced gene expression changes across EC clusters in (**c**) visceral and (**d**) subcutaneous AT. Any gene showing a |log(FC)| > 0.1 in any cluster was used to collate the list of genes used for these analyses. The numbers show the Pearson’s r-value for each comparison. (**e**) BioPlanet-annotated pathways enriched in DEGs in the cap2 versus cap1 population in visceral AT. Data are expressed as -log_10_(p-adj). Genes significantly enriched (p-adj < 0.05) in cap2 versus cap1 ECs were used for this analysis. (**f**) Heatmap showing obesity-associated gene expression changes in select ECM components. a – arteries + arterioles (art), c – capillaries, v – veins + venules (ven). Art, capillary and ven ECs were defined using markers outlined in Extended Data Fig. 2. (**g**) Heatmap showing gene expression changes in the ‘Integrin signaling’ pathway in cap ECs. Cap ECs were defined using markers outlined in Extended Data Fig. 2. (**h-i**) Quantification of (**h**) angiogenic and (**i**) proliferating ECs in each of the 7 tissues. Top genes enriched in angiogenic and proliferating ECs in the AT are provided in Extended Data Fig. 3a, b and Supplementary Tables 3–6. Data were analyzed using a two-sided χ^2^-test. (**j**) Obesity-associated changes in the expression of genes associated with angiogenesis and proliferation in visceral and sc AT capillary ECs. (**k-l**) BioPlanet-annotated terms significantly enriched in genes (**k**) upregulated and (**l**) downregulated in LECs in visceral and sc AT. Data are expressed as -log_10_(p-adj). Top 100 (**k**) up- and (**l**) downregulated genes, ranked by log(FC), were used for these analyses. p-adj indicates adjustments for multiple comparisons using the Benjamini-Hochberg method, FC – fold change. Differential expression from scRNA-seq data is expressed on a natural log (log_e_) scale. Source data

We focused first on AT ECs, given that they had the highest number of DEGs (Fig. 1e). Visceral AT and subcutaneous AT had 10 and 12 EC clusters, respectively (Fig. 2a–d and Extended Data Fig. 3a–d). The cap2 population, which had enriched expression of genes encoding extracellular matrix (ECM) and integrin interactions, was elevated in visceral AT from obese mice versus controls (Fig. 2b and Extended Data Fig. 3e). Consistently, cap ECs in AT, but not other organs, showed obesity-associated upregulation of genes related to integrin signaling, focal adhesions and ECM components (Fig. 2e,f and Extended Data Fig. 3f,g). Among these, a particularly strong upregulation of *Itgb1* mRNA and integrin-β1 protein levels was observed in both visceral AT and subcutaneous AT ECs (Fig. 2f–h).

Obesity induced a greater than threefold increase in angiogenic and proliferating ECs in the AT (Fig. 2i and Extended Data Fig. 3h,i), consistent with the expansion and remodeling of AT in obesity. Notably, 11% of ECs in subcutaneous AT from obese mice displayed an angiogenic or proliferative phenotype (Fig. 2i). Genes known to regulate EC proliferation, migration and maturation, namely *Rhoc*, *Tmsb10*, *Dll4*, *Sox4*, *Col4a1*, *Col4a2*, *Kdr*, *Flt1*, *Acvrl1* and *Itgb1* (refs. ^19–21^), showed obesity-associated changes in AT cap ECs (Extended Data Fig. 3j).

In comparison to other organs, our FACS isolation procedure yielded the highest number of lymphatic ECs (LECs) in the visceral AT (Fig. 2j). Visceral and subcutaneous LECs displayed an obesity-associated upregulation of the RAGE pathway, integrins and ECM–receptor interactions, while a downregulation in the brain-derived neurotrophic factor (BDNF), interleukin-5 (IL-5), IL-2 and AP1 networks was observed (Extended Data Fig. 3k,l).

#### Liver ECs

**Extended Data Fig. 4:**
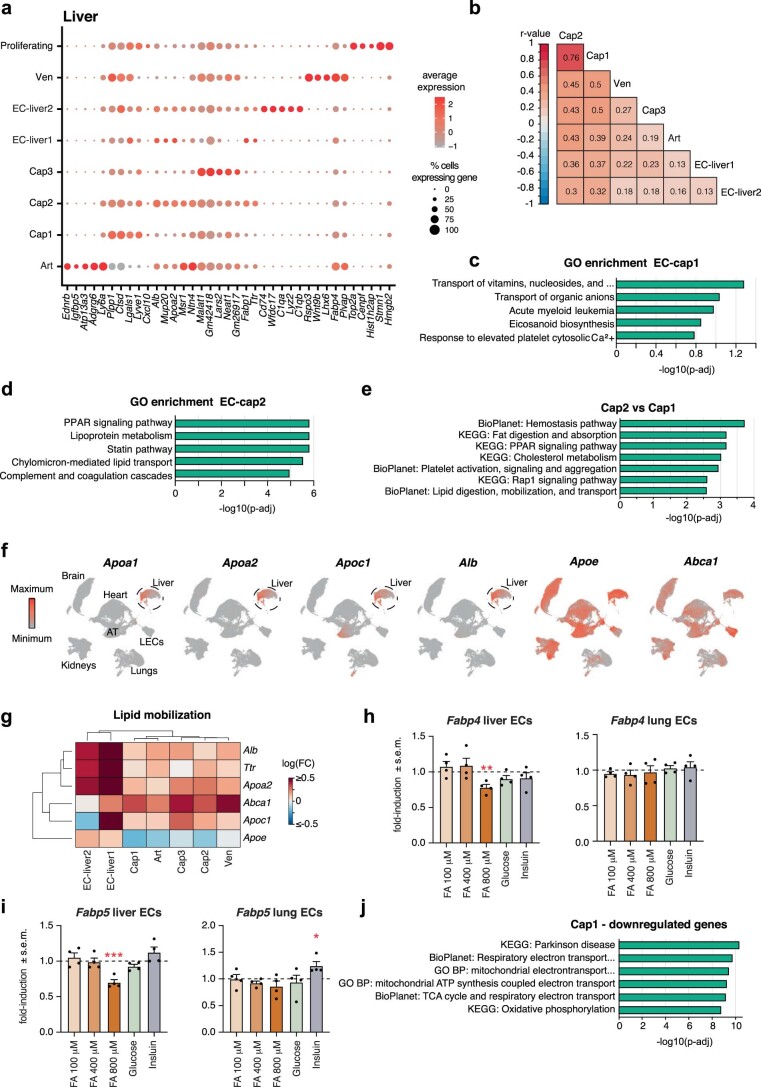
Liver ECs activate lipid mobilization networks in obesity. (**a**) Top 5 enriched genes in liver EC clusters. (**b**) Comparison of obesity-associated gene expression changes across EC clusters in liver. Any gene showing a |log(FC)| > 0.1 in any cluster was used to collate the list of genes used for these analyses. The numbers show the Pearson’s r-value for each comparison. (**c**) BioPlanet-annotated pathways significantly enriched in the liver cap1 population relative to other capillary ECs. Data are expressed as -log_10_(p-adj). (**d**) BioPlanet-annotated pathways significantly enriched in the liver cap2 population relative to other capillary ECs. Data are expressed as -log_10_(p-adj). (**e**) KEGG and BioPlanet terms most significantly enriched in cap2 versus cap1 population. Data are expressed as -log_10_(p-adj). Genes significantly enriched (p-adj < 0.05) in the cap2 population versus cap1 were used for this analysis. (**f**) UMAPs showing expression of *Apoa1*, *Apoa2*, *Apoc1*, *Alb, Apoe* and *Abca1* in ECs across all organs. (**g**) Heatmap showing induction of lipid mobilization genes in liver EC clusters relative to chow controls. (**h-i**) Induction of (**h**) *Fabp4* and (**i**) *Fabp5* mRNA expression in response to 100 µM, 400 µM and 800 µM free fatty acids (FA), as well as 30 mM glucose and 10 µg per ml insulin in primary mouse liver and lung ECs. Data were standardized against BSA-treated controls and are presented as mean ± SEM. N = 4 replicates per group, with ECs for each replicate derived from a different animal. Expression data were standardized to *Gapdh* and *Rplp0*. (**h**) **p = 0.002; (**i**) ***p = 0.0003, *p = 0.021 (two-sided Student’s t-test). Each condition was compared against BSA-controls. (**j**) KEGG, BioPlanet and gene ontology – biological process (GO BP) terms enriched in genes downregulated in the liver cap1 population in obesity. Data are expressed as -log_10_(p-adj). p-adj indicates adjustments for multiple comparisons using the Benjamini-Hochberg method, FC – fold change. Differential expression from scRNA-seq data is expressed on a natural log (log_e_) scale. Source data

The liver is a major processor of lipids and is highly impacted by obesity^22^. We observed eight EC clusters in liver, including two liver-specific EC subtypes, EC-liver1 and EC-liver2 (Fig. 2k and Extended Data Fig. 4a,b). The cap2 cluster was increased in obesity (Fig. 2l) and was enriched for lipid-processing pathways, such as PPAR signaling, fat digestion and absorption (Extended Data Fig. 4c–e).

The expression of lipid mobilization genes was highly enriched in ECs from liver versus other organs and showed obesity-associated upregulation in liver ECs, particularly in the EC-liver1 and EC-liver2 populations (Fig. 2m and Extended Data Fig. 4f,g). Similarly, obesity triggered increased expression of the liver-specific fatty acid transporter *Fabp1* in hepatic cap ECs and of fatty acid transporters *Fabp4*, *Cd36*, *Fabp5*, *Dbi* and *Lpl* in art and ven ECs from AT (Fig. 2n,o). *Fabp1*, but not *Fabp4* or *Fabp5* mRNA, increased in primary mouse liver ECs following 24-h treatment with 800 µM free fatty acids (Fig. 2p and Extended Data Fig. 4h,i), which is similar to the concentration of fatty acids found in the serum of individuals with obesity^23,24^. Consistent with the predicted binding of PPARα and IRF3 in the promoter region of *Fabp1* (Fig. 2q), the fatty acid-mediated increase in *Fabp1* mRNA was mitigated by an inhibitor of TBK1 and IKKε (MRT67307), which are upstream activators of IRF3, and an inhibitor of PPARα (GW6471; Fig. 2q). This suggests that obesity-associated *Fabp1* upregulation is promoted by fatty acids via the PPARα and TBK1–IKKε–IRF3 pathways. In contrast to the upregulation of lipid-handling genes, mitochondrial respiration genes were downregulated with obesity in liver cap ECs (Extended Data Fig. 4j). Together, these analyses suggest an obesity-induced shift in lipid handling and metabolic networks in liver ECs.

Gene Ontology (GO) analysis revealed enriched expression of platelet activation and aggregation genes in liver cap2 ECs (Extended Data Fig. 4d,e). To determine if this was associated with increased platelet adhesion to cap ECs, we stained liver sections with the activated platelet marker CD62P. Indeed, livers from obese mice showed increased staining for CD62P compared to those from chow controls (Fig. 2r), consistent with increased platelet activation and adhesion to liver ECs.

#### Cardiac ECs

**Fig. 3:**
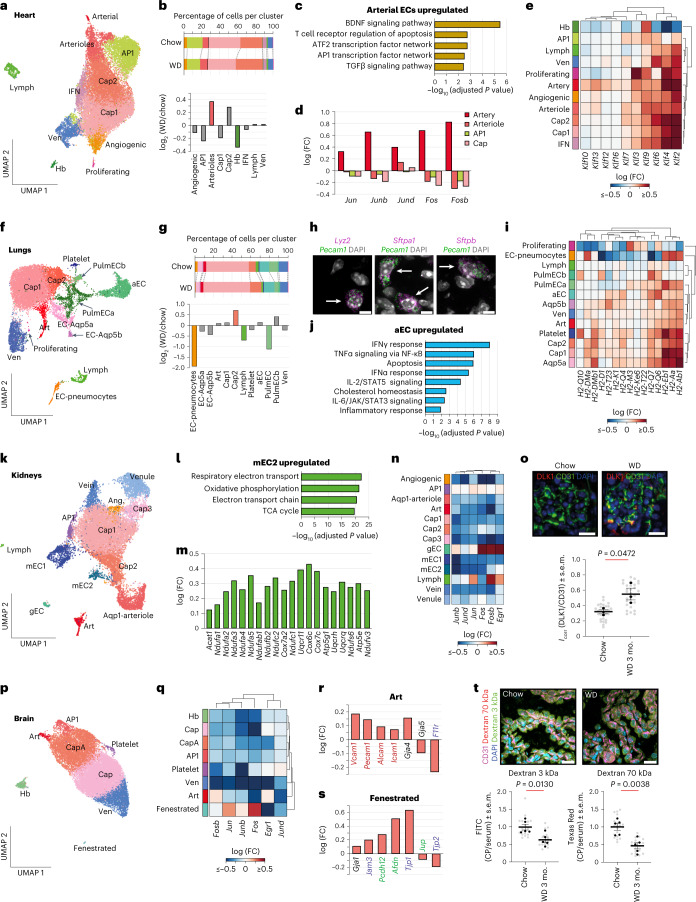
Obesity triggers deregulation of metabolic and inflammatory networks in subsets of cardiac, lung, kidney and brain ECs. **a**, UMAP clustering of cardiac ECs. **b**, Shifts in cardiac EC populations. Populations showing a log_2_ (WD/chow) > 0.3 change are highlighted in color. **c**, BioPlanet-annotated pathways upregulated in cardiac arterial ECs in obesity. The top 100 genes, ranked by fold change, were used for these analyses. **d**, Obesity-associated changes in the expression of AP1 transcription factor subunits. **e**, Obesity-associated gene expression changes in KLF-family transcription factors. **f**, UMAP clustering of lung ECs. **g**, Obesity-associated shifts in lung EC clusters. Populations showing a log_2_ (WD/chow) > 0.5 change are highlighted in color. **h**, FISH images showing overlap of typical pneumocyte markers (*Lyz2*, *Sftpa1* and *Sftpb*) and EC marker (*Pecam1*). Double-positive cells are marked with arrows. Data were reproduced in three chow and three obese animals; scale bars, 5 µm. **i**, Changes in the expression of histocompatibility 2 (*H2*) genes in obesity. **j**, MSigDB-annotated pathways upregulated in aEC in obesity. The top 100 genes, ranked by fold change, were used for these analyses. **k**, UMAP clustering of kidney ECs; Ang, angiogenic. **l**, BioPlanet-annotated pathways upregulated in mEC2 cells in obesity. The top 100 genes, ranked by fold change, were used for these analyses; TCA, tricarboxylic acid. **m**, Top metabolic DEGs in mEC2 cells in obesity. **n**, Obesity-associated changes in the expression of AP1 transcription factor subunits. **o**, Quantification of DLK1 in gECs; *n* = 3 animals per group (black dots) and *n* = 10 images per animal (gray dots); scale bars, 20 µm. **p**, UMAP clustering of brain ECs. **q**, Obesity-associated changes in the expression of AP1 transcription factor subunits. **r**,**s**, Gene expression changes in select leukocyte adhesion (marked in red), tight junction (blue), adherens junction (green) and gap junction genes (black) in art (**r**) and fenestrated ECs (**s**) in obesity. **t**, Uptake of dextran dyes in the choroid plexus (CP); *n* = 5 animals per group (black dots); *n* = 4 sections per animal (gray dots); scale bars, 20 µm. Data in **o** and **t** are presented as mean ± s.e.m. and were analyzed using a two-sided Student’s *t*-test. The adjusted *P* value indicates adjustments for multiple comparisons using the Benjamini–Hochberg method (**c**, **j** and **l**). Source data

**Extended Data Fig. 5:**
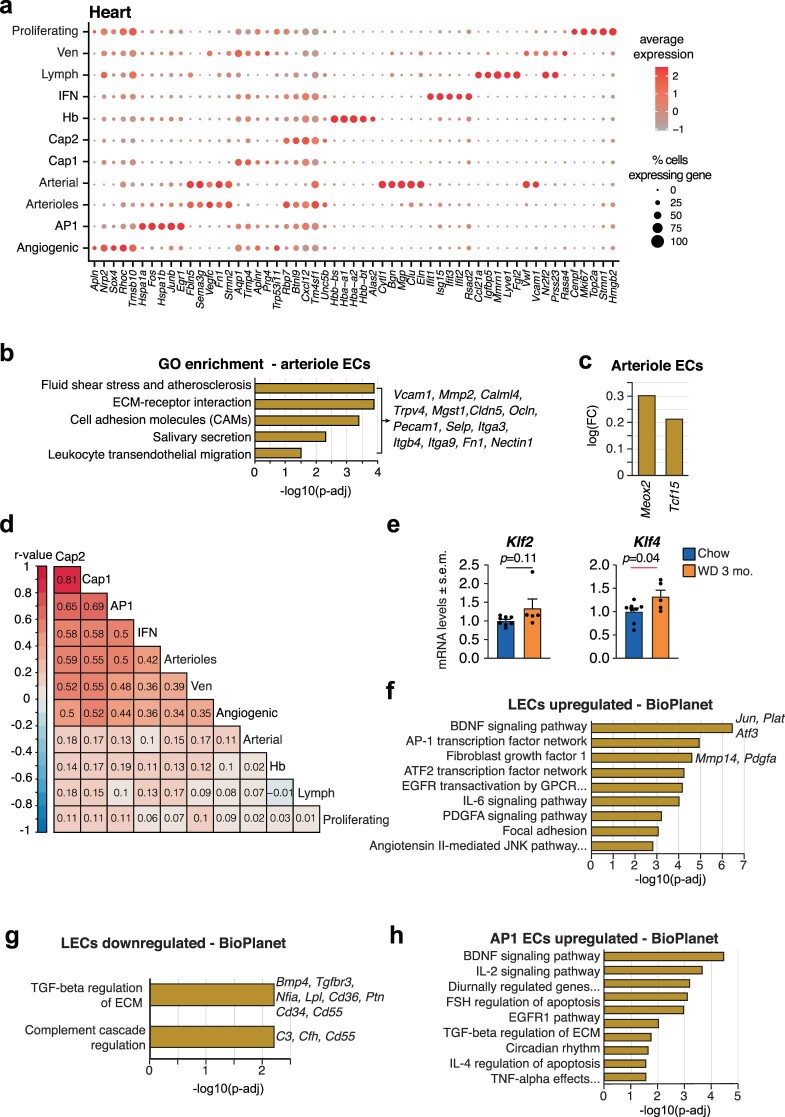
Leukocyte migration and inflammation networks are activated by obesity in cardiac ECs. (**a**) Top 5 enriched genes in each of the heart EC clusters. (**b**) GO terms for genes significantly enriched in arteriole ECs relative to other cardiac ECs. Data are expressed as -log_10_(p-adj). Exemplary genes found across all listed GO categories are provided. Genes significantly enriched (p-adj < 0.05) in the arteriole EC population relative to other cardiac ECs were used for this analysis. (**c**) Obesity-associated gene expression changes in *Meox2* and *Tcf15* in cardiac arteriole ECs. (**d**) Comparison of obesity-associated gene expression changes across cardiac EC clusters. Any gene showing a |log(FC)| > 0.1 in any cluster was used to collate the list of genes used for these analyses. Pearson’s r-value for each comparison is provided. (**e**) Relative *Klf2* and *Klf4* mRNA levels in bulk FACS-isolated CD31^+^ CD45^−^ cells from hearts of control and obese animals. N = 8 animals in chow group; 5 in WD group. Data were standardized to *Rplp0* expression and analyzed using a two-sided Student’s t-test. Data are presented as mean ± SEM. (**f-g**) BioPlanet-annotated terms significantly (**f**) upregulated and (**g**) downregulated in cardiac LECs in obesity. Data are expressed as -log_10_(p-adj). Top 100 (**f**) up- and (**g**) downregulated genes, ranked by log(FC), were used for these analyses. Exemplary genes for select pathways are listed. (**h**) BioPlanet annotated pathways significantly upregulated in AP1 ECs in obesity. Data are expressed as -log_10_(p-adj). Top 100 upregulated genes, ranked by log(FC), were used for these analyses. p-adj indicates adjustments for multiple comparisons using the Benjamini-Hochberg method, FC – fold change. Differential expression from scRNA-seq data is expressed on a natural log (log_e_) scale. Source data

Obesity promotes atherosclerosis and thus is a major risk factor for myocardial infarction^25^. Our scRNA-seq analyses of mouse hearts revealed 11 EC clusters, including interferon-high ECs (IFN-ECs), hemoglobin-positive ECs (Hb-ECs) and AP1 transcription factor-high ECs (AP1-ECs; Fig. 3a and Extended Data Fig. 5a).

We observed an increase in the arteriole EC subtype with obesity (Fig. 3b). Genes associated with shear stress and atherosclerosis, ECM–receptor interaction and leukocyte transendothelial migration showed enriched expression in arteriole ECs compared to other ECs in the heart (Extended Data Fig. 5b). Furthermore, arteriole ECs showed obesity-induced expression of *Meox2* and *Tcf15* (Extended Data Fig. 5c), which activate a fatty acid uptake program^26^.

Unexpectedly, obesity-induced DEGs in heart arterial ECs were markedly different than in arteriole, cap and ven ECs, which were relatively similar (Extended Data Fig. 5d). Obesity led to increased expression of the TGFβ signaling pathway and AP1 transcription factor network in arterial ECs (Fig. 3c). The AP1 transcription factor subunit genes *Jun*, *Junb*, *Jund*, *Fos* and *Fosb* were upregulated in arterial ECs in obesity but not in arteriole ECs, AP1-ECs or cap ECs (Fig. 3d). Given the association between the AP1 transcription factor and atherosclerosis^27^, our data suggest that cardiac artery ECs are particularly prone to developing atherosclerosis in obesity.

Cardiac EC clusters showed obesity-induced expression of the Krüppel-like (KLF) transcription factor genes *Klf2*, *Klf3*, *Klf4*, *Klf6* and *Klf9* (Fig. 3e and Extended Data Fig. 5e), which play an important role in vascular inflammation and response to shear stress^28^. Furthermore, cardiac LECs showed an upregulation of the BDNF, FGF1 and ATF2 transcription factor networks (Extended Data Fig. 5f,g), while the AP1 EC cluster showed an induction in inflammatory pathways, including IL-2, IL-4 and tumor necrosis factor-α (TNFα) signaling (Extended Data Fig. 5h). Induction of these particular inflammatory networks was not detected in other cardiac EC populations.

#### Lung ECs

**Extended Data Fig. 6:**
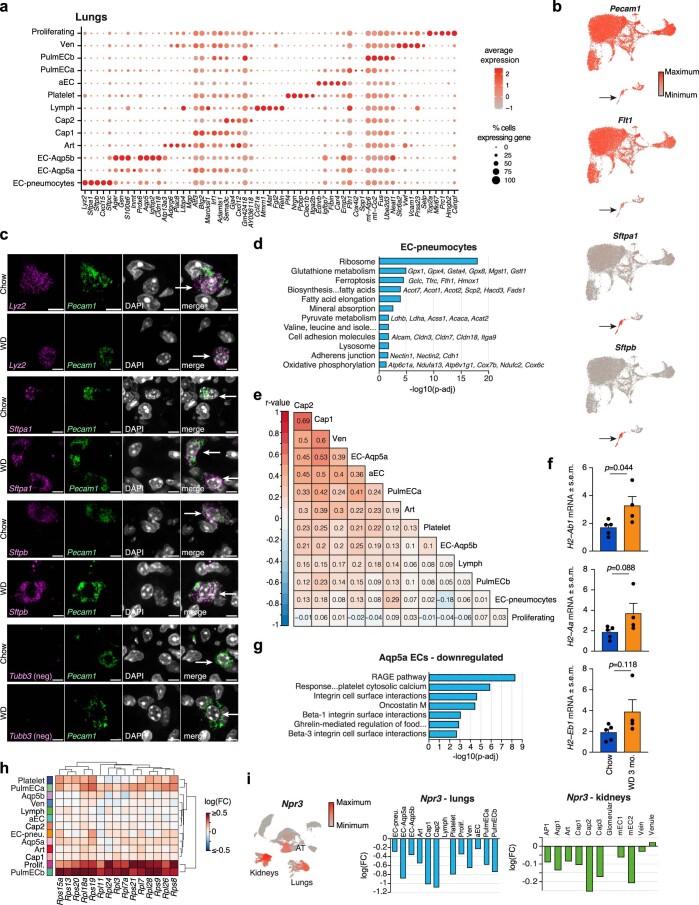
Obesity induces inflammatory networks in lung ECs. (**a**) Top 5 enriched genes in each of the lung EC clusters. (**b**) UMAPs showing the co-expression of EC markers *Pecam1* and *Flt1* with pneumocyte markers *Sftpa1* and *Sftpb*. Arrows indicate the population showing overlap of pneumocyte and EC markers. (**c**) Representative fluorescence *in situ* hybridization (FISH) images showing the colocalization of pneumocyte markers (*Lyz2, Sftpa1*, *Sftpb*) and endothelial marker *Pecam1*. *Tubb3* is provided as a negative control, as it is typically not expressed in the lungs. White arrows indicate the double positive cells. The data were reproduced in 3 control and 3 WD fed animals. Scale bars equal 5 µm. (**d**) KEGG curated pathways enriched in the EC-pneumocyte population relative to other lung ECs. Data are expressed as -log_10_(p-adj). Genes significantly enriched (p-adj < 0.05) in the EC-pneumocyte population relative to other lung ECs were used for this analysis. (**e**) Comparison of obesity-associated gene expression changes across lung EC clusters. Any gene showing a |log(FC)| > 0.1 in any cluster was used to collate the list of genes used for these analyses. The numbers presented are Pearson’s r-value for each comparison. (**f**) Expression of histocompatibility genes *H2-Ab1*, *H2*-*Aa* and *H2-Eb1* in bulk FACS-isolated CD31^+^ CD45^−^ cells from lungs of control and obese animals. N = 5 animals in chow group; 4 in WD group. Data were standardized to *Hsp90ab1* and *Gapdh* expression. Data are presented as mean ± SEM and were analyzed via a Student’s t-test. (**g**) BioPlanet curated pathways downregulated in Aqp5a ECs in obesity. Data are expressed as -log_10_(p-adj). Top 100 downregulated genes, ranked by log(FC), were used for these analyses. (**h**) Heatmap showing obesity-induced gene expression changes in select ribosomal genes across lung EC clusters. (**i**) UMAP of *Npr3* expression across organs. Bar graphs represent obesity-associated changes in *Npr3* expression in lung and kidney ECs. p-adj indicates adjustments for multiple comparisons using the Benjamini-Hochberg method, FC – fold change. Differential expression from scRNA-seq data is expressed on a natural log (log_e_) scale. Source data

Analysis of lung ECs from control and obese animals revealed 13 clusters, including two populations of pulmonary ECs (pulmECa and pulmECb), two populations of Aqp5-positive ECs (EC-Aqp5a and EC-Aqp5b) and a population of platelet marker gene-positive (platelet) ECs and alveolar ECs (aEC; Fig. 3f and Extended Data Fig. 6a). The pulmECa and LEC populations were reduced in obesity, whereas the cap2 population increased (Fig. 3g).

We also detected a small cluster that coexpresses typical EC markers (*Pecam1* and *Flt1*) and the surfactant genes *Sftpa1*, *Sftpb* and *Sftpc*, which are markers of pneumocytes, the epithelial cells involved in gas exchange that line the alveoli (Extended Data Fig. 6a,b). We termed this population ‘EC-pneumocytes’ and confirmed its presence via fluorescence in situ hybridization (FISH), which revealed cells that coexpress the EC marker *Pecam1* together with *Lyz2*, *Sftpa1* and *Sftpb* (Fig. 3h and Extended Data Fig. 6c). Relative to other ECs, EC-pneumocytes were enriched for the expression of ribosomal and metabolic genes (Extended Data Fig. 6d). The EC-pneumocyte population was substantially reduced in obese animals (Fig. 3g).

Obesity-induced DEGs were different across EC subtypes in the lung (Extended Data Fig. 6e). The lung cap populations (cap1 and cap2) showed activation of inflammatory networks with an induction of histocompatibility genes in obese animals (Fig. 3i and Extended Data Fig. 6f). Moreover, obesity induced the activation of IFN, TNFα, IL-2 and IL-6 signaling in the aEC population (Fig. 3j). These data suggest that obesity generally increases the inflammatory state in lung ECs.

The EC-Aqp5a population showed a reduction in the RAGE pathway and integrin interactions in obesity, while the pulmECa, pulmECb and proliferating ECs showed an upregulation in ribosomal gene expression (Extended Data Fig. 6g,h).

*Npr3* was the most impacted DEG across all lung EC subtypes. *Npr3* encodes the natriuretic peptide receptor 3, which removes natriuretic peptides from the blood to maintain sodium balance, diuresis, blood pressure and vascular tone^29^*. Npr3* was strongly expressed in kidney and lung ECs and to a lesser extent in AT ECs (Extended Data Fig. 6i). Similar to lung ECs, kidney ECs showed a global downregulation of *Npr3* in obesity, albeit to a lesser extent. This finding suggests that sodium balance and vascular tone in the body are likely impacted by deregulation of lung and kidney ECs in obesity.

#### Kidney ECs

**Extended Data Fig. 7:**
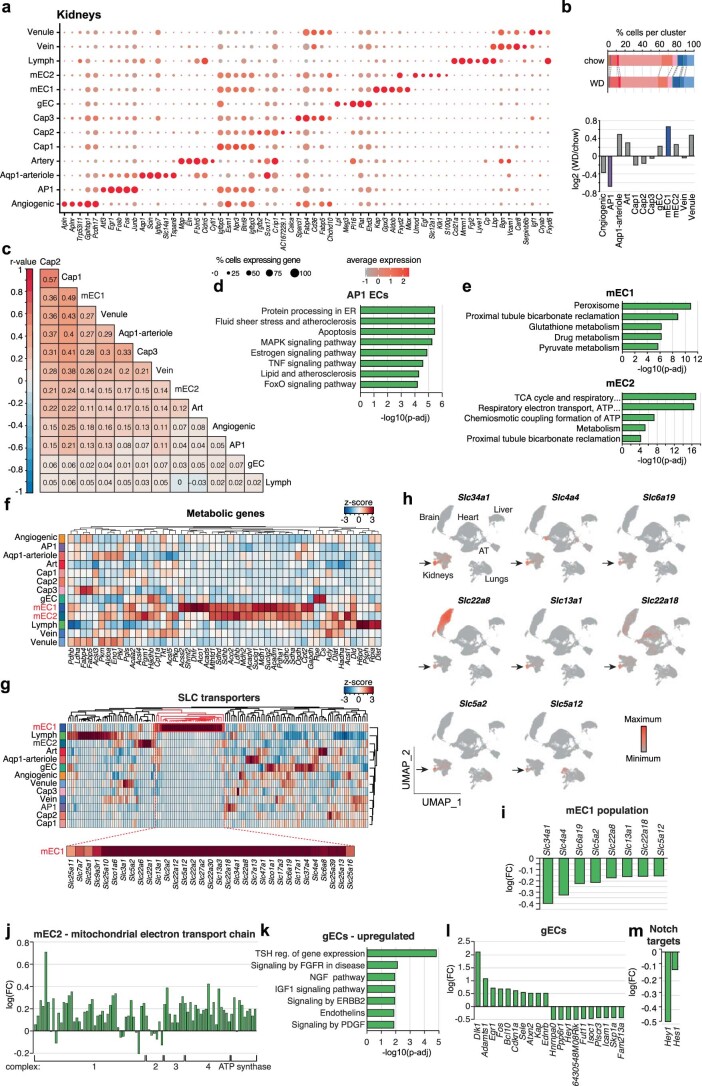
Obesity induces metabolic changes and downregulation of solute transporters in kidney ECs. (**a**) Top 5 enriched genes in each of the kidney EC clusters. mEC – medullary ECs, gEC – glomerular ECs. (**b**) Obesity-associated shifts in kidney EC clusters. Populations showing a change of log_2_(WD/chow) > 0.5 in obesity are highlighted in color. (**c**) Comparison of obesity-associated gene expression changes across renal EC subtypes. Any gene showing a |log(FC)| > 0.1 in any cluster was used to collate the list of genes used for these analyses. Pearson’s r-value for each comparison is provided. (**d**) KEGG curated pathways significantly enriched in AP1 ECs versus other kidney ECsGenes significantly enriched (p-adj < 0.05) in AP1 ECs were used for these analyses. (**e**) KEGG pathways enriched in the mEC1 and mEC2 clusters relative to other kidney ECs. Genes significantly enriched (p-adj < 0.05) in mEC1 and mEC2, relative to other kidney EC populations, were used for these analyses. (**f**) Enrichment in the expression of metabolic genes across kidney EC clusters. Z-scores of average gene expression per cluster are presented. (**g**) Enrichment of genes encoding SLC transporters across kidney EC clusters. SLC transporters enriched in the mEC1 population are highlighted in red and indicated below the heatmap. (**h**) UMAPs showing the enrichment of *Slc34a1*, *Slc4a4*, *Slc6a19*, *Slc5a2*, *Slc22a8*, *Slc13a1*, *Slc22a18* and *Slc5a12* in renal mEC1 cells. The mEC1 population is marked by the black arrows. (**i**) Obesity-associated gene expression changes in SLC transporters in the mEC1 population. (**j**) Changes in the expression of mitochondrial respiratory genes in the mEC2 population in obesity. Respiratory complex identities are marked at the bottom. (**k**) BioPlanet curated pathways upregulated in glomerular ECs (gECs) in obesity. Top 100 downregulated genes, ranked by log(FC), were used for these analyses. (**l**) Top differentially expressed genes, ranked by *p* value, in gECs. (**m**) Obesity-associated changes in the expression of Notch target genes *Hey1* and *Hes1* in gECs. p-adj indicates adjustments for multiple comparisons using the Benjamini-Hochberg method, FC – fold change, gEC – glomerular EC, mEC – medullary EC. Differential expression from scRNA-seq data is expressed on a natural log (log_e_) scale. Source data

We identified a total of 13 renal EC clusters, including glomerular ECs (gECs), two populations of medullary ECs (mECs), *Aqp1*-positive ECs (Aqp1 arteriole ECs) and AP1 transcription factor-high ECs (AP1-ECs; Fig. 3k and Extended Data Fig. 7a,b). Apart from cap ECs, there was little correlation in obesity-induced DEGs across kidney EC subtypes (Extended Data Fig. 7c).

Obesity led to a reduction in the kidney AP1 EC population (Extended Data Fig. 7b). Relative to other ECs, AP1-ECs were enriched for genes involved in protein processing in the endoplasmic reticulum as well as MAPK, TNF and FOXO signaling networks (Extended Data Fig. 7d).

By contrast, obesity increased the size of the mEC1 population, which, like the mEC2 population, showed enriched expression of genes related to metabolic pathways and bicarbonate reclamation (Extended Data Fig. 7b,e,f). mEC1 cells were highly enriched for solute transporters, many of which were not expressed in ECs of other organs (Extended Data Fig. 7g,h). The mEC1 population showed an obesity-induced downregulation of transporters of major ions (*Slc34a1*, *Slc4a4*, *Slc22a8*, *Slc13a1*, *Slc22a18* and *Slc5a12*), neutral amino acids (*Slc6a19*) and glucose (*Slc5a2*; Extended Data Fig. 7i). By contrast, obesity induced the upregulation of mitochondrial respiration and metabolic genes in mEC2 cells (Fig. 3l,m and Extended Data Fig. 7j). Thus, obesity has distinct impacts on the mEC compartment in the kidney.

In contrast to cardiac artery ECs (Fig. 3d), renal ECs generally showed reduced expression of AP1 transcription factor subunit genes *Jun*, *Junb*, *Jund*, *Fos*, *Fosb* and *Egr1* in obesity (Fig. 3n). However, gECs showed increased expression of *Fos*, *Fosb* and *Egr1* and increased expression of FGF receptor, nerve growth factor and IGF1 signaling networks in obesity (Fig. 3n and Extended Data Fig. 7k). In gECs, *Dlk1*, which encodes an inhibitor of Notch signaling and angiogenesis^30^, was the strongest upregulated gene in obesity (Extended Data Fig. 7l), and levels of DLK1 protein were increased twofold in obesity (Fig. 3o). Consistently, reduced expression of Notch target genes *Hey1* and *Hes1* was detected in gECs (Extended Data Fig. 7m). These data uncover unique impacts of obesity on the cortical (art, cap and ven), mEC and gEC populations in the kidney.

#### Brain ECs

**Extended Data Fig. 8:**
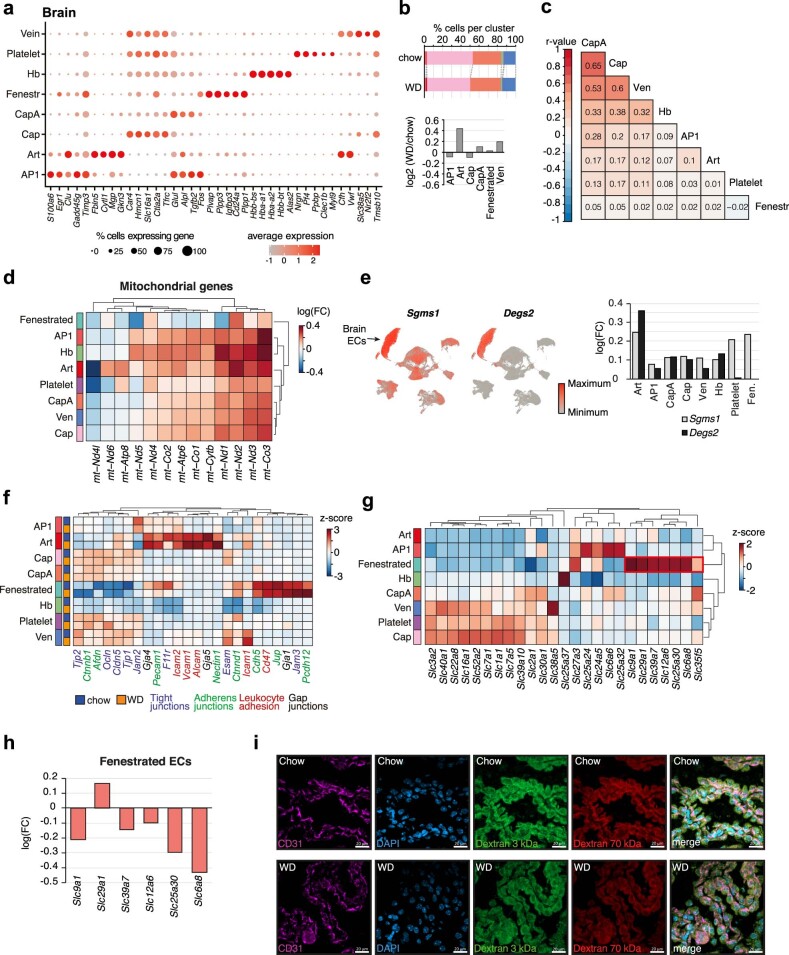
Brain ECs show obesity-induced changes in metabolic, cell junction and solute carrier genes. (**a**) Top 5 enriched genes in each of the neural EC clusters. (**b**) Shifts in neural EC clusters. Quantification of the shifts is provided in the lower bar graph, with changes expressed on a log2 scale. (**c**) Comparison of obesity-associated gene expression changes across neural EC subtypes. Any gene showing a |log(FC)| > 0.1 in any cluster was used to collate the list of genes used for these analyses. Pearson’s r-value for each comparison is provided. (**d**) Heatmap depicting obesity-induced gene expression changes in mitochondrial encoded members of the electron respiration chain. (**e**) UMAPs depicting enrichment of *Sgms1* and *Degs2* mRNA in ECs across all organs. Obesity-associated gene expression changes in neural ECs are shown. (**f**) Enrichment (z-scores) of tight junctions (blue), adherent junctions (green), gap junctions (black) and leukocyte adhesion molecules (red) across neural EC clusters. (**g**) Heatmap showing the enrichment (z-scores) of SLC transporters across neural EC clusters. The *Slc* genes enriched in fenestrated EC population are indicated by the red box. (**h**) Gene expression changes in select *Slc* genes in fenestrated ECs in obesity. (**i**) Representative mages of Dextran 3 kDa (FITC) and Dextran 70 kDa (Texas red) in the choroid plexus of obese and control animals. ECs were immunostained with CD31 (purple). All images were taken with the same exposure and laser power. Quantifications are provided in Fig. 2r. N = 5 animals per group. Scale bars represent 20 µm. FC – fold change. Differential expression from scRNA-seq data is expressed on a natural log (log_e_) scale. Source data

Defects in neural vasculature are closely related to cognitive decline and neurodegeneration^31^. Analysis of brain ECs revealed eight major clusters, including fenestrated ECs, Hb-ECs, platelet-ECs and AP1-ECs (Fig. 3p and Extended Data Fig. 8a,b). Obesity-induced DEGs were similar in ven and cap brain ECs, whereas other EC populations, and fenestrated ECs in particular, showed unique DEGs (Extended Data Fig. 8c).

Similar to kidney ECs, neural ECs showed downregulation of AP1 transcription factor subunit genes *Fos*, *Fosb*, *Jun*, *Junb*, *Jund* and *Egr1* in obesity (Fig. 3q). By contrast, obesity led to upregulation of mitochondrial-encoded subunits of the electron transport chain across EC clusters, suggesting a general shift in the metabolic profile of brain ECs (Extended Data Fig. 8d). Relative to other EC clusters, gene expression changes in AP1 transcription factor subunits and metabolic genes were less pronounced in fenestrated ECs (Fig. 3q and Extended Data Fig. 8d). *Sgms1* and *Degs2*, which encode proteins required for the production of sphingolipids, showed highly enriched expression in brain ECs and were upregulated in obesity, particularly in the art cluster (Extended Data Fig. 8e).

Relative to other brain ECs, art ECs showed enriched expression of several leukocyte adhesion genes (Extended Data Fig. 8f), and *Vcam1*, *Pecam1*, *Alcam* and *Icam1* were upregulated in art ECs in obesity (Fig. 3r). These data suggest that leukocyte adhesion and transendothelial migration increase in brain art in obesity.

Fenestrated ECs are typically found in the choroid plexus. Cell junction proteins showed enriched expression in fenestrated ECs (Extended Data Fig. 8f), and *Jam3*, *Pdch12*, *Afdn* and *Tjp1* were upregulated in obesity in fenestrated ECs (Fig. 3s). Several solute transporters showed enriched expression in fenestrated ECs and were downregulated in obesity (Extended Data Fig. 8g,h). These data suggest that obesity reduces transport across the choroid plexus. Consistently, in vivo tracing with fluorescent dextran dyes showed reduced dye uptake by fenestrated ECs in the choroid plexus of obese animals (Fig. 3t and Extended Data Fig. 8i). Given that defective EC transport and function in the choroid plexus is linked with memory deficiency^32^ and neurodegeneration^33,34^, our data uncover a potential link between obesity and deregulation of neural homeostasis.

### Sustained obesity exacerbates gene dysregulation in ECs

**Fig. 4:**
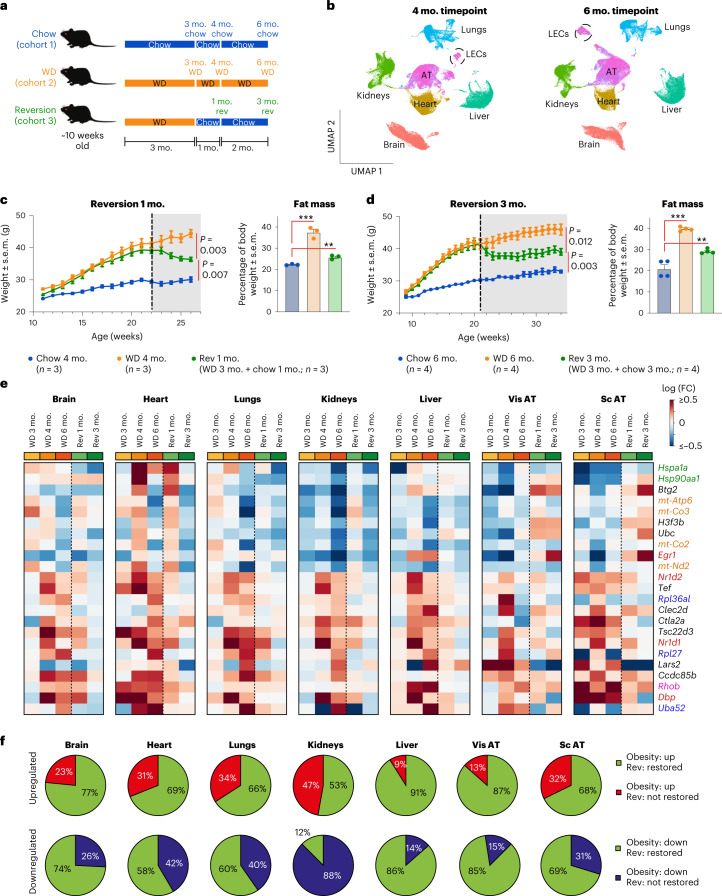
Switching obese animals to a healthy diet modifies trajectory of animal weight, fat mass and the EC transcriptome. **a**, Experimental design. For the reversion group (cohort 3), animals were fed a WD for 3 months and switched to a chow diet. Data for the 3-month chow and WD timepoints are from Figs. 1–3; rev, reversion. **b**, UMAPs of all ECs analyzed at the 4-month and 6-month timepoints after filtering out low-quality and non-ECs. **c**,**d**, Weights and percent body fat mass of animals analyzed at the 4-month (**c**) and 6-month (**d**) timepoints. The dotted line indicates when the WD was switched to a chow diet in reversion animals. Data are presented as mean ± s.e.m.; ***P = 0.0005 and **P = 0.0029 (**c**); ***P = 7.56 × 10^−5^ and **P = 0.0062 (**c**,**d**; two-sided Student’s *t*-test). **e**, Heat map representing genes most commonly impacted in cap ECs across organs in obesity and after reversion diet. The list of genes was generated based on the 6-month WD timepoint. The following genes are marked: translation-related genes (blue), transcription regulators (red), stress response genes (green), electron respiratory chain genes (orange) and signaling molecules (pink). Data were standardized to the appropriate chow control at each timepoint. **f**, Proportion of up- and downregulated genes in cap ECs that retain the obesity transcriptional profile or change their trajectory toward a healthier profile in the reversion group (cohort 3). Gene expression changes after 6 months of a WD were compared to the 3-month reversion timepoint. Data for both WD and reversion groups were standardized to chow controls at each timepoint. ‘Restored’ indicates genes in the reversion group that show expression levels more similar to the chow versus WD group. Source data

**Extended Data Fig. 9:**
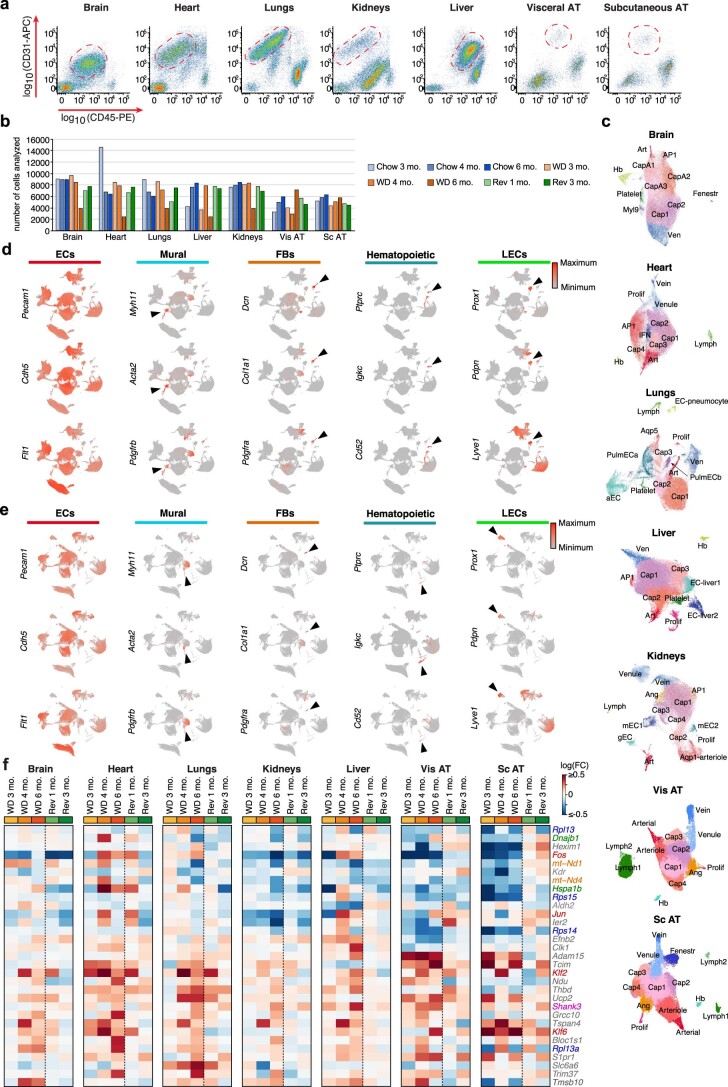
Impact of diet reversion on ECs. (**a**) Representative FACS plots showing enrichment of ECs based on PECAM1 (CD31) and CD45 staining. ECs from the heart, brain, lungs, kidneys and liver were enriched at the 4- and 6-month timepoints using CD31 magnetic beads prior to staining and FACS. (**b**) Number of ECs analyzed at each timepoint and in each experimental group after filtering out low quality and non-ECs. (**c**) UMAPs of ECs combined from all timepoints showing distinct clusters in each organ. Markers described in Extended Data Figs. 1–8 were used to define these populations. These markers were originally obtained from published scRNA-seq datasets^11,13,16–18^. (**d**) UMAPs showing the presence of typical EC, mural, fibroblast (FB), hematopoietic and lymphatic EC (LEC) markers at the 4-month time point (4-month chow, 4-month WD, 1-month reversion groups). The arrows indicate the positive population in each category. (**e**) UMAPs showing the presence of typical EC, mural, fibroblast (FB), hematopoietic and lymphatic EC (LEC) markers at the 6-month time point (6-month chow, 6-month WD, 3-month reversion groups). The arrows indicate the positive population in each category. (**f**) Select genes showing similar deregulation across all organs in obesity. The 6-month WD timepoint was used to select these genes. Expression is shown relative to the chow control at each timepoint. Following genes are marked: translation-related genes (blue), transcription regulators (red), stress response genes (green), electron respiratory chain-related genes (orange), and signaling molecules (pink). FC – fold change. Differential expression from scRNA-seq data is expressed on a natural log (log_e_) scale. Source data

To examine how dietary intervention affects obesity and EC phenotypes, we monitored three cohorts of mice: mice fed a chow diet for 6 months (cohort 1, chow diet), mice fed a WD for 6 months (cohort 2, sustained WD) and mice fed a WD for 3 months followed by a chow diet for 3 months (cohort 3, reversion diet; Fig. 4a). ECs were isolated by FACS at the 4- and 6-month timepoints and analyzed by scRNA-seq (Fig. 4a,b and Extended Data Fig. 9a). After switching diets, cohort 3 showed an initial reduction in body weight and fat mass, which stabilized at intermediate levels that were between those observed in animals fed exclusively a WD or chow diet (Fig. 4c,d). These data suggest that reversion to a chow diet only partially restores body weight and fat mass.

After filtering, a total of 376,293 ECs were compiled across eight experimental groups for further analysis (Extended Data Fig. 9b–e; chow 3-month and WD 3-month groups analyzed above, chow 4- and 6-month, WD 4- and 6-month and reversion 1- and 3-month groups).

First, we identified obesity-induced DEGs in cap ECs that were shared across the seven tissues. We focused on cap ECs because they had the highest number of DEGs across organs. The gene encoding ubiquitin A52 ribosomal protein fusion product (*Uba52*), which is important for protein translation^35^, showed the strongest upregulation across cap ECs in sustained obesity (cohort 2; Fig. 4e). Similarly, genes encoding circadian clock regulators (*Dbp*, *Nr1d1* and *Nr1d2*), KLF transcription factors (*Klf2* and *Klf6*) and cell signaling molecules (*Rhob* and *Shank3*) were also upregulated in sustained obesity (cohort 2) but not in the reversion group (cohort 3; Fig. 4e and Extended Data Fig. 9f). Genes related to the stress response, including heat-shock proteins (*Hspa1a*, *Hsp90aa1*, *Dnajb1* and *Hspa1b*), AP1 transcription factor subunits (*Fos* and *Jun*), *Egr1*, VEGF receptor 2 (*Kdr*) and electron respiratory chain-related genes (*mt-Atp6*, *mt-Co3*, *mt-Co2*, *mt-Nd2*, *mt-Nd1* and *mt-Nd4*) were downregulated in sustained obesity (cohort 2; Fig. 4e and Extended Data Fig. 9f). The expression of these genes was more similar to control levels following a reversion diet (cohort 3). Thus, a diverse set of genes, including transcriptional regulators, cell signaling molecules, stress response and metabolic genes, become systemically deregulated in cap ECs with sustained obesity.

### Organ-specific EC responses to dietary intervention

Next, we investigated what proportion of gene expression changes observed in sustained obesity could be prevented by dietary intervention. We defined a gene as restored if its expression in reversion diet-fed animals (cohort 3) was closer to that observed in chow controls (cohort 1) than in mice fed exclusively the WD (cohort 2). We found that liver cap ECs were the most responsive to dietary intervention, with ~88% of DEGs in sustained obesity remedied by dietary intervention (Fig. 4f). Moreover, 60–85% of DEGs in cap ECs from AT, heart, lungs and brain were remedied by dietary intervention (Fig. 4f). Strikingly, kidney cap ECs were less responsive to dietary interventions; 47% of upregulated genes and 88% of downregulated genes were unaffected by the reversion diet compared to the exclusive WD (Fig. 4f). These data suggest that, of the organ-specific ECs, kidney ECs are the most vulnerable to obesity.

#### AT ECs

**Fig. 5:**
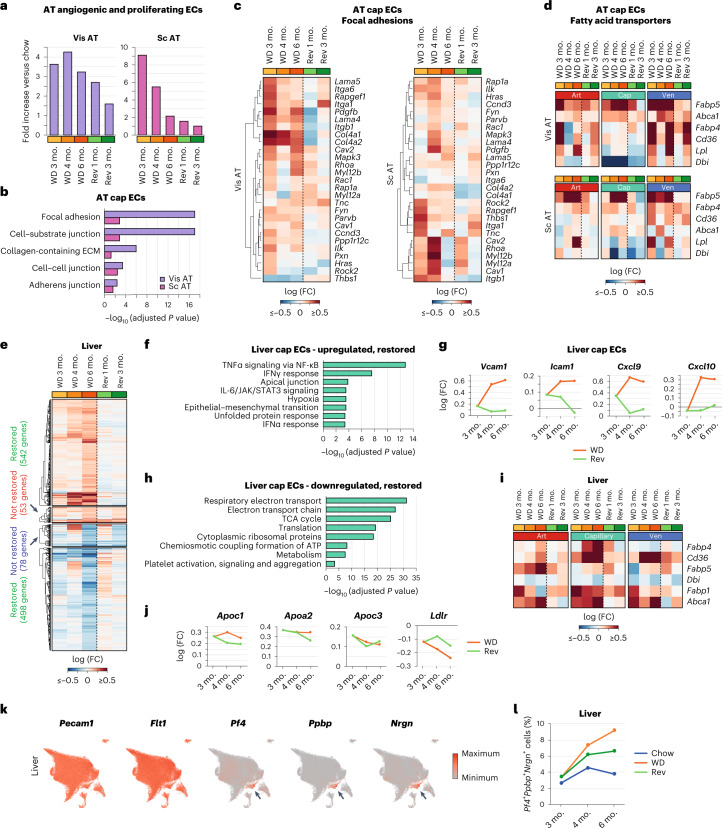
Improved trajectories of ECM components in AT ECs and of metabolic and inflammatory networks in liver ECs in the reversion group. **a**, Changes in the proportion of angiogenic and proliferating ECs in visceral AT and subcutaneous AT in WD-treated and reversion cohorts. Data are presented relative to chow controls at each timepoint. **b**, GO cellular component pathways upregulated in AT cap ECs in obesity, which switch toward chow levels in the reversion group. **c**, Gene expression changes in focal adhesion genes in cap ECs in visceral and subcutaneous AT. **d**, Gene expression changes in fatty acid transporters in art, cap and ven ECs in the visceral and subcutaneous AT. **e**, DEGs (adjusted *P* value of <0.05 and | log (FC) | > 0.1) in hepatic cap ECs at the 6-month timepoint. Genes that show a restored transcriptional profile in the reversion group are indicated. **f**, BioPlanet-annotated pathways upregulated in cap ECs in obesity (adjusted *P* value of < 0.05 and log (FC) > 0.1), which are restored toward chow levels in the reversion group. **g**, Expression of *Vcam1*, *Icam1*, *Cxcl9* and *Cxcl10* in WD and reversion groups in liver cap ECs. **h**, BioPlanet-annotated pathways downregulated in hepatic cap ECs in obesity (adjusted *P* value of <0.05 and log (FC) < 0.1), which are restored toward healthy chow levels in the reversion group. **i**, Gene expression changes in fatty acid transporters in liver art, cap and ven ECs. **j**, Expression of *Apoc1*, *Apoa2*, *Apoc3* and *Ldlr* in WD and reversion groups in liver cap ECs. **k**, UMAPs showing coexpression of endothelial markers (*Pecam1* and *Flt1*) and platelet markers (*Pf4*, *Ppbp* and *Nrgn*) in the liver EC-platelet population, which is marked by the black arrow. **l**, Percentage of ECs positive for *Pf4*, *Ppbp* or *Nrgn* in liver in chow, WD and reversion cohorts at each timepoint. The adjusted *P* value indicates adjustments for multiple comparisons using the Benjamini–Hochberg method (**b**, **f** and **h**). Source data

**Extended Data Fig. 10:**
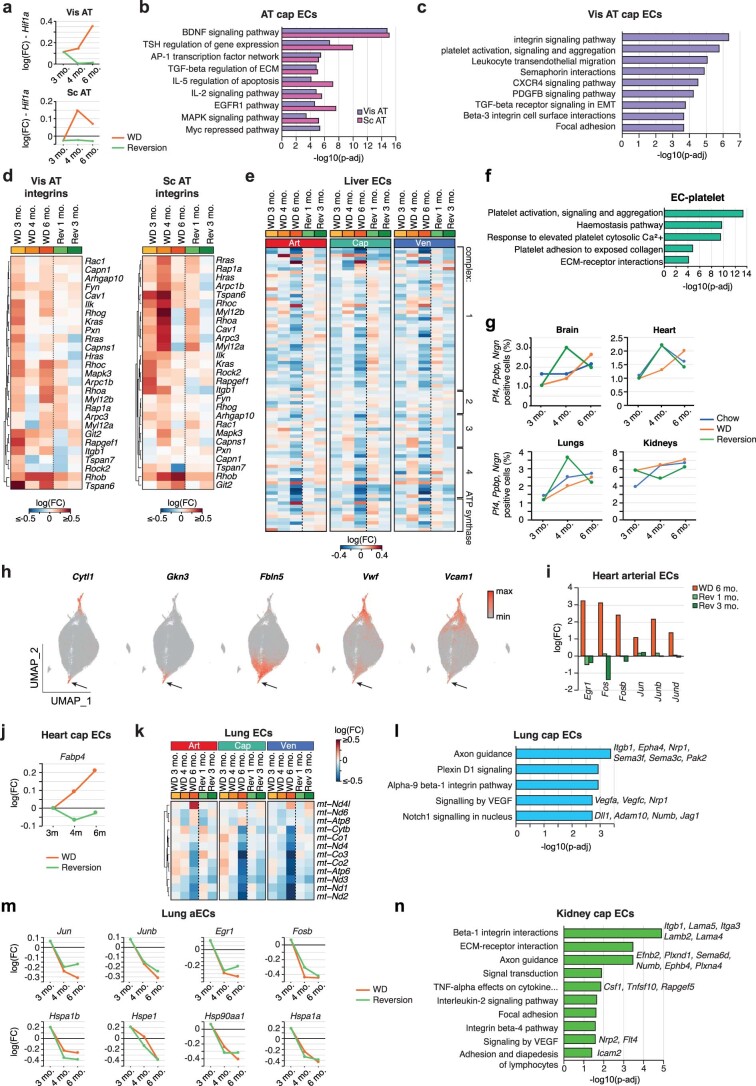
Changes in trajectories of EC transcriptome following a reversion diet. (**a**) Changes in the expression levels of *Hif1a* in AT cap ECs. (**b**) BioPlanet pathways enriched in visceral and sc AT cap ECs. Analysis was done on genes significantly downregulated by sustained obesity (6 m WD, p-adj < 0.05, |log(FC)| > 0.1), which recover towards control levels in the dietary reversion group. (**c**) BioPlanet terms upregulated by sustained obesity (6 m WD, p-adj < 0.05, |log(FC)| > 0.1) in visceral AT ECs, which recover towards control levels in the dietary reversion group. (**d**) Gene expression changes of integrin signaling network in cap ECs of visceral and subcutaneous (sc) AT. (**e**) Gene expression changes in mitochondrial respiratory network in liver art, cap and ven EC clusters. Respiratory complex identities are marked on the right side. (**f**) BioPlanet terms enriched in the brain EC-platelet population relative to other ECs. Genes significantly enriched (p-adj < 0.05) in the EC-platelet population were used for these analyses. (**g**) Quantification of platelet marker-positive ECs in the brain, heart, lungs, and kidneys as a proportion of all ECs in the respective organ. (**h**) UMAPs showing enrichment of large vessel and arterial markers in heart ECs. The arrow denotes arterial ECs, where all the tested markers overlap. (**i**) Gene expression changes in AP1 transcription factor subunits in cardiac arteries. (**j**) Expression of *Fabp4* in heart cap ECs. (**k**) Obesity-associated gene expression changes in mitochondria-encoded genes in lung art, cap and ven EC populations. (**l**) BioPlanet terms significantly upregulated by sustained obesity (6 m WD, p-adj < 0.05, |log(FC)| > 0.1) in lung cap ECs, which do not recover towards control levels in the dietary reversion group. (**m**) Changes in expression levels of AP1 transcription factor subunits and *Hsp* genes in the lung aEC population. (**n**) BioPlanet pathways upregulated by sustained obesity (6 m WD, p-adj < 0.05, |log(FC)| > 0.1) in the kidney cap population, which do not recover towards control levels in the dietary reversion group. p-adj indicates adjustments for multiple comparisons using the Benjamini-Hochberg method, FC – fold change. Differential expression from scRNA-seq data is expressed on a natural log (log_e_) scale. Source data

Consistent with remodeling of the AT during obesity, the WD promoted EC angiogenesis and proliferation (Fig. 5a). However, the proportion of angiogenic and proliferating ECs was reduced in subcutaneous AT with sustained obesity compared to the earlier 3- and 4-month WD timepoints (Fig. 5a). This is consistent with defective angiogenesis in clinical obesity^36^. Furthermore, the gene encoding hypoxia-inducible factor 1-α (*Hif1a*) was upregulated in cap ECs in visceral AT at the 6-month timepoint (Extended Data Fig. 10a). These observations suggest that AT ECs display a more diseased state with sustained obesity.

Sustained obesity led to the upregulation of integrin and focal adhesion networks in cap ECs, and this effect was mitigated by the reversion diet (Fig. 5b,c and Extended Data Fig. 10b–d). AT cap ECs displayed a reduction in BDNF, TSH and AP1 transcription factor networks with sustained obesity, which was not observed in the 3-month reversion group (Extended Data Fig. 10b). Among fatty acid transporters, *Fabp5* showed the strongest obesity-induced upregulation in AT ECs (Fig. 5d). The expression of fatty acid transporters was partially normalized in the 3-month reversion group in AT ECs (Fig. 5d).

#### Liver ECs

Inflammatory networks were induced in liver cap ECs from mice fed a WD for 6 months but not in mice fed the reversion diet (Fig. 5e,f). In particular, the expression of *Vcam1*, *Icam1*, *Cxcl9* and *Cxcl10* was restored to normal levels after 3 months of chow on the reversion diet (Fig. 5g).

We found that sustained obesity exacerbated the reduced expression of mitochondrial respiration subunits across liver ECs, but this was mitigated by dietary reversion (Fig. 5h and Extended Data Fig. 10e). Consistent with increased circulating lipids and fatty acids in obesity, a 6-month WD induced increased expression of fatty acid and lipid transporters *Cd36*, *Fabp4*, *Fabp5* and *Abca1* in liver ECs, which was partially restored by the reversion diet (Fig. 5i).

Although most DEGs in liver cap ECs were restored in the reversion group (Fig. 5e), the expression levels of *Apoc1*, *Apoa2* and *Apoc3* remained high (Fig. 5j). Similarly, low-density lipoprotein receptor (LDLR) *Ldlr* mRNA levels remained low in liver cap ECs in the dietary reversion group (Fig. 5j). The lack of improvement in *Apo* and *Ldlr* gene expression is important, as high levels of APO proteins and low levels of LDLR increase the risk of cardiovascular disease^37,38^.

After 3 months on the WD, platelet activation and adhesion to liver cap ECs was already increased (Fig. 2r). We observed a cluster of ECs that displayed typical platelet transcripts (Fig. 5k and Extended Data Fig. 10f), and sustained obesity increased the size of this population in the liver but not in other organs (Fig. 5l and Extended Data Fig. 10g). The frequency of platelet-positive ECs in the liver was only partly restored in mice fed the reversion diet compared to those sustained on the WD. As increased platelet activation and adhesion are related to tissue and endothelial damage^39^, our observations suggest that the liver endothelium is prone to damage in obesity, and this can be partially mitigated by weight loss.

#### Cardiac ECs

**Fig. 6:**
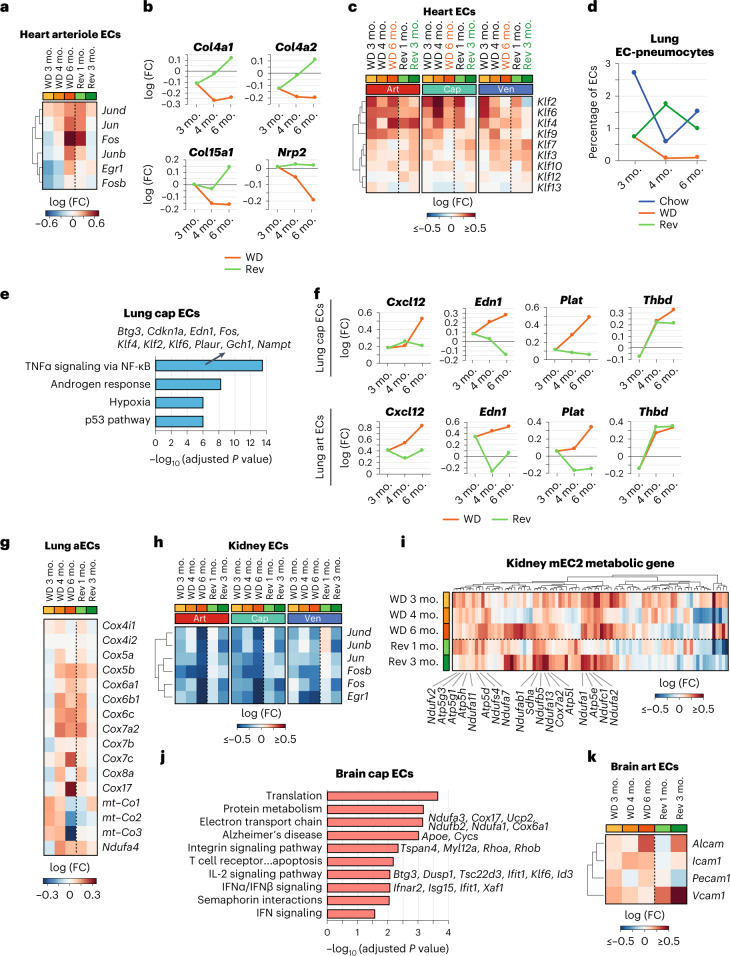
Partial improvement in trajectories of obesity-induced gene expression changes in heart, lung, kidney and brain ECs by a reversion diet. **a**, Changes in the gene expression levels of AP1 transcription factor subunits in arteriole ECs in obesity and reversion conditions. **b**, DEGs associated with ECM organization, including *Col4a1*, *Col4a2*, *Col15a1* and *Nrp2*, in cardiac cap ECs. **c**, Gene expression changes in *Klf* genes in cardiac art, cap and ven ECs of obese and reversion animals. **d**, Quantification of the EC-pneumocyte population as a proportion of all lung ECs. **e**, MSigDB-curated pathways upregulated in obesity in lung cap ECs (adjusted *P* value of <0.05 and log (FC) > 0.1), which show improved trajectory in the diet reversion group. **f**, Expression levels of select inflammation-associated genes upregulated in lung cap and art ECs in obesity. **g**, Gene expression changes in members of the fourth mitochondrial respiratory chain complex in the aEC population. **h**, Gene expression changes in AP1 transcription factor subunits in kidney art, cap and ven ECs of obese and reversion animals. **i**, Differential expression of genes encoding mitochondrial respiration subunits in the kidney mEC2 population. Select genes are indicated. Data were standardized to the chow control group at each timepoint. **j**, BioPlanet-annotated pathways upregulated in brain cap ECs in obesity (adjusted *P* value of <0.05 and log (FC) > 0.1), showing an improved trajectory in the reversion group. **k**, Expression of select leukocyte adhesion genes in brain art ECs. Adjusted *P* values in **e** and **j** indicate adjustments for multiple comparisons using the Benjamini–Hochberg method. Source data

The AP1 transcription factor subunit genes *Jun*, *Junb*, *Jund*, *Fos* and *Fosb* were already upregulated in heart arterial ECs after 3 months on the WD and were further upregulated in mice fed the WD for 6 months (Extended Data Fig. 10h,i). These genes were also upregulated in arteriole ECs following 6 months on the WD (Fig. 6a). The expression of AP1 subunits was restored to control levels following 3 months on the reversion diet, which was particularly striking in arterial ECs (Extended Data Fig. 10h,i). These data suggest that the risk of atherosclerosis in cardiac arteries can be at least partially mitigated by an improved diet.

Cardiac cap ECs showed reduced expression of ECM organization genes with sustained obesity, which was mitigated by the reversion diet (Fig. 6b). Similarly, sustained obesity increased the expression of the fatty acid transporter *Fabp4* in heart cap ECs, and this was not observed in the reversion group (Extended Data Fig. 10j).

Obesity upregulated the expression of *Klf2*, *Klf4*, *Klf6* and *Klf9* at all timepoints in heart art, cap and ven ECs (Fig. 6c). Interestingly, a reversion diet reduced the expression of *Klf* genes to control levels in cap and ven ECs but not in cardiac art ECs. Given that KLF factors are activated in response to stress^28^, our data suggest that art ECs in the heart continue to experience some level of stress despite an improved diet.

#### Lung ECs

The EC-pneumocyte population in the lungs showed an approximately threefold reduction after 3 months on the WD. This population was further depleted after 4 and 6 months of the WD but recovered strongly in the diet reversion group (Fig. 6d).

Similar to the 3-month timepoint, GO analysis revealed induction of inflammatory networks in lung cap ECs following 6 months of a WD but not in the diet reversion group (cohort 3; Fig. 6e). Consistently, expression of inflammatory genes *Cxcl12*, *Edn1*, *Plat* and *Thbd* increased in lung cap and art ECs in the WD group (Fig. 6f). Of these, *Cxcl12*, *Edn1* and *Plat* showed reduced expression in the diet reversion group, whereas *Thbd* mRNA remained high. Sustained obesity reduced the levels of mitochondrial-encoded transcripts in lung art, cap and ven EC populations, which was attenuated by dietary reversion (Extended Data Fig. 10k). By contrast, ECM genes, VEGF signaling members *Vegfa* and *Vegfc* and Notch1 signaling genes *Dll1*, *Jag1* and *Numb* were upregulated by sustained obesity in lung cap ECs but were not attenuated by a reversion diet (Extended Data Fig. 10l).

The aEC cluster showed an upregulation of genes encoding mitochondrial respiration subunits and, in particular, the fourth respiratory complex with sustained obesity, but not in the dietary reversion group (Fig. 6g). Notably, despite not being impacted by a 3-month WD, AP1 subunits and heat-shock protein (*Hsp*) genes *Hsp1a*, *Hsp1b*, *Hsp1e* and *Hsp90aa1* were downregulated in the lung aEC population with sustained obesity and in the dietary reversion group (Extended Data Fig. 10m), suggesting that obesity primes the deregulation of stress response genes in this population.

#### Kidney ECs

Kidney ECs were most vulnerable to obesity and showed the least improvement after a reversion diet (Fig. 4f). Kidney ECs showed a global downregulation of AP1 transcription factors with sustained obesity that was not restored by the reversion diet (Fig. 6h). Similarly, kidney cap ECs upregulated ECM components, namely integrins, laminin subunits and ephrins, with sustained obesity, and the expression of these genes did not improve after weight loss (Extended Data Fig. 10n). The kidney mEC2 population showed obesity-induced upregulation of diverse metabolic genes, including glycolysis and respiration genes, even in mice fed the reversion diet (Fig. 6i). This result is particularly striking, as upregulation of metabolic networks in kidney mECs is associated with cellular stress, such as hyperosmolarity^13^, suggesting that short-term or moderate obesity is sufficient to induce a stress state in kidney mECs.

#### Brain ECs

Sustained obesity led to increased expression of genes associated with protein translation, electron transport chain, Alzheimer’s disease (for example, *Apoe* and *Cycs*), integrin and IFN signaling (Fig. 6j). The expression of these networks was mitigated by a reversion diet. Genes associated with leukocyte migration, such as *Alcam*, *Icam1*, *Pecam1* and *Vcam1*, were induced in brain art ECs by sustained obesity (Fig. 6k). However, only *Icam1* and *Pecam1* showed lower expression levels with the reversion diet (Fig. 6k).

### Disease-associated genes are induced by obesity

To identify obesity-induced DEGs in ECs that can increase the risk of vascular dysfunction, we integrated our 6-month WD and 6-month chow datasets with the NHGRI-EBI GWAS database^40^. The NHGRI-EBI database in manually curated and quality controlled and contains all published GWAS studies that meet the quality threshold^40^. We focused on genetic risk loci associated with disorders having vascular pathologies, such as coronary artery disease, atherosclerosis, heart failure, hypertension, stroke, Alzheimer’s disease and bipolar disorder.

**Fig. 7:**
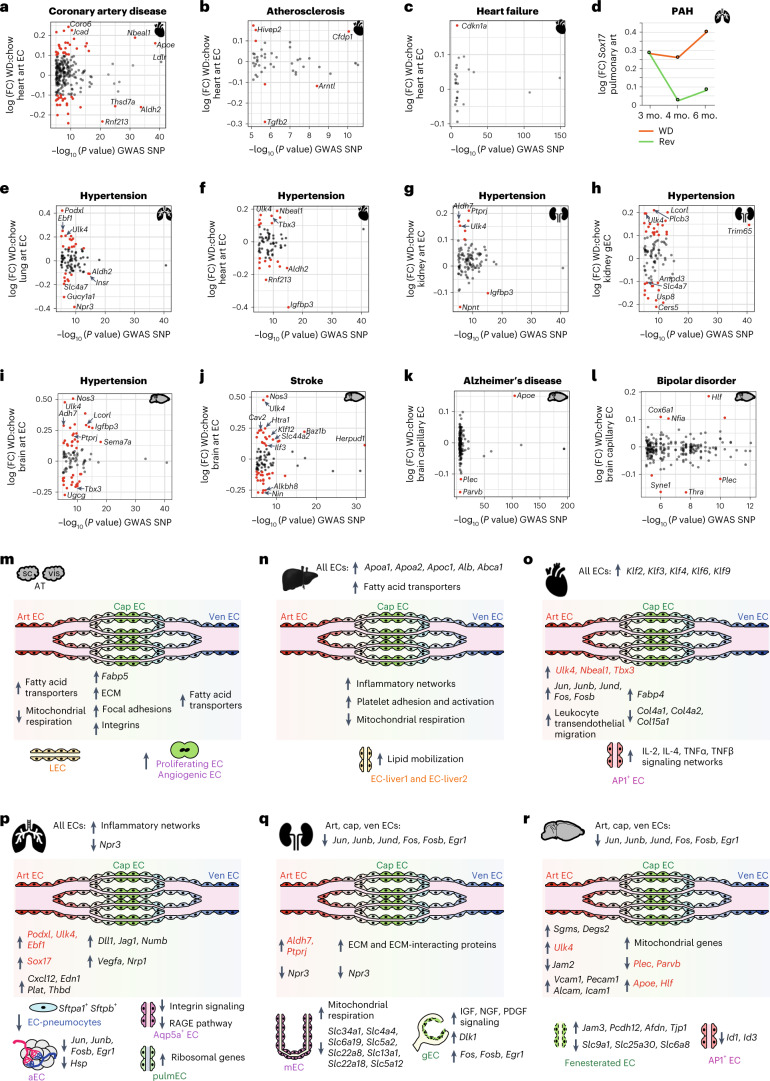
Integration of human GWAS data reveals vascular disease risk genes that are induced by obesity. **a**, Comparison of known high-risk variants (identified via GWAS) for coronary artery disease with obesity-induced gene expression changes in heart art ECs at the 6-month timepoint. **b**, Comparison of known high-risk variants for atherosclerosis with obesity-induced gene expression changes in heart art ECs at the 6-month timepoint. **c**, Comparison of known high-risk variants for heart failure with obesity-induced gene expression changes in heart art ECs at the 6-month timepoint. **d**, Expression levels of *Sox17*, the most significant genetic risk factor for pulmonary arterial hypertension (PAH), in pulmonary art ECs in obesity and after reversion. **e**–**i**, Comparison of known high-risk variants for hypertension with obesity-induced gene expression changes in lung art (**e**), heart art (**f**), kidney art (**g**), kidney gEC (**h**) and brain art (**i**) ECs at the 6-month timepoint. **j**, Comparison of known high-risk variants for stroke with obesity-induced gene expression changes in brain art ECs at the 6-month timepoint. **k**, Comparison of known high-risk variants for Alzheimer’s disease with obesity-induced gene expression changes in brain cap ECs at the 6-month timepoint. **l**, Comparison of known high-risk variants for bipolar disorder with obesity-induced gene expression changes in brain cap ECs at the 6-month timepoint. **m**–**r**, Summary of the most prominent obesity-induced gene expression changes in EC populations identified in this study. Changes in EC populations of the AT (**m**), liver (**n**), heart (**o**), lungs (**p**), kidneys (**q**) and brain (**r**) are provided. Genes indicated in red are high-risk genes for the development of vascular pathologies and overlap with human GWAS studies. The *x* axes in **a**–**c** and **e**–**l** represent the –log_10_ (*P* value) of disease-associated SNPs assigned to a gene, while the *y* axes in **a**–**l** represent the log (FC) of the marked gene in obesity. Genes with | log (FC) | > 0.1 are highlighted in red. Select candidate genes are labeled. SNPs associated with each disease were obtained from the NHGRI-EBI GWAS database. Source data

Gene loci associated with coronary artery disease, including neurobeachin-like 1 (*NBEAL1)* and *APOE*, were upregulated in heart art ECs in obesity (Fig. 7a). Interestingly, genetic variants of *NBEAL1*, a gene that is poorly understood at the functional level, also significantly increases the risk of atherosclerosis development in young individuals and promotes the risk of myocardial infarction^41,42^. These observations strongly suggest *NBEAL1* as an obesity-induced candidate gene that could increase the risk of disease in cardiac art ECs.

Further genes upregulated in cardiac art ECs in obesity, including *Hivep2* and *Cfdp1*, were associated with atherosclerosis (Fig. 7b), while *CDKN1A*, the gene encoding the senescence factor p21, was associated with heart failure (Fig. 7c). As age and EC senescence are important risk factors for heart failure^43^, the induction of *Cdkn1a* suggests that obesity may induce an aging phenotype in heart arterial ECs, which in turn promotes heart failure.

Genetic variants in *SOX17* were recently reported as the strongest risk factor for pulmonary arterial hypertension^44^. Obesity led to the upregulation of *Sox17* in lung art ECs, which, importantly, was mitigated by the reversion diet (Fig. 7d). *ULK4* is a significant genetic risk factor for systemic hypertension and was upregulated with obesity in lung art, heart art, kidney art, brain art and gECs (Fig. 7e–i). ULK4 is a serine–threonine kinase that plays an important role in brain development^45^. The function of ULK4 in ECs is not well understood, but *ULK4* genetic variants are associated with aortic disease^46^. *Ulk4* was the only high-risk hypertension gene that was systemically upregulated in art ECs with obesity. Other hypertension-associated genes impacted by obesity included *Podxl* and *Ebf1* upregulated in lung art ECs, *Nbeal1* and *Tbx3* upregulated in heart art ECs and *Igfbp3* with reduced expression in heart, kidney and brain art ECs (Fig. 7e–i).

Defects in neural vasculature are associated with numerous diseases, including stroke, Alzheimer’s disease and bipolar disorder^47–49^. Genetic variants of *ULK4*, *CAV2*, *HTRA1* and *KLF12* are all associated with increased risk of stroke and the orthologous genes were upregulated in brain art ECs in obesity (Fig. 7j). Genetic variants of *APOE* are the strongest known risk factor for Alzheimer’s disease^50^, and *Apoe* was upregulated in brain cap ECs in obesity (Fig. 7k). Genes encoding the cytoskeleton-interacting proteins PLEC and PARVB are risk factors for Alzheimer’s disease, and genetic variants of *PLEC* and *SYNE1* are high-risk alleles for bipolar disorder (Fig. 7k,l). All three genes were downregulated in brain cap ECs with obesity. Reduced levels of *Plec*, *Parvb* or *Syne1* can adversely impact EC shape and EC permeability^51–53^. Given that dysregulation of vascular permeability is an early hallmark of neurodegeneration^31^, our data suggest that changes in cytoskeleton networks in brain cap ECs in obesity promote a diseased state.

## Discussion

Our study systematically investigated the impact of obesity on ECs. Our scRNA-seq analyses of ~375,000 ECs revealed that obesity differentially impacts EC subtypes across AT, liver, heart, lungs, kidneys and brain (Fig. 7m–r). We show that dietary intervention can partially mitigate changes in body weight, fat mass and EC transcriptomes, suggesting weight loss and improved metabolic health has an overall positive effect on the endothelium. Our work identified vascular dysfunction risk genes, including *Sox17*, *Ulk4*, *Nbeal1*, *Cdkn1a* and *Plec*, as obesity-regulated genes in the endothelium. These data implicate these genes in the pathophysiology of obesity-induced vascular defects associated with diseases, such as atherosclerosis, heart failure, neurodegeneration, stroke and pulmonary hypertension. Our data represent a valuable resource for the community and are publicly available through an interactive website at https://obesity-ecatlas.helmholtz-muenchen.de.

Our findings suggest that obesity primarily affects ECs in an organ- and subtype-specific manner rather than in a global manner (Fig. 7m–r). This finding is in agreement with the unique physiological functions of blood vessels in different organs and the organ-specific expression profiles of vascular cells^11,12,54^. Nevertheless, why different EC subpopulations show opposing responses to the same physiological trigger, namely obesity, remains unclear. For instance, genes encoding AP1 transcription factor subunits were upregulated specifically in heart arterial ECs but not in other heart EC populations (Fig. 3d). Furthermore, AP1 transcription factor expression was repressed in kidney and brain ECs in obesity (Fig. 3n,q). In a similar manner, fatty acids were able to upregulate *Fabp1* expression in liver ECs but not lung ECs (Fig. 2p). We speculate that these organ-specific responses of ECs are due to a primed epigenetic state, which reflects the functionality of the respective organ. For instance, as the liver is a major metabolic hub, liver ECs are likely programmed to transport fatty acids to support liver function. By contrast, lungs are not known to play a major role in lipid or fatty acid metabolism and, therefore, are unlikely to possess the machinery to transport fatty acids in the presence or absence of metabolic stress. Consistently, EC subtypes are likely to respond differently to metabolic triggers, such as increased fatty acids, glucose or insulin, depending on the signaling networks active in the EC subtype and the primed epigenetic state of target genes. Future studies integrating analysis of obesity-induced epigenomic changes with our scRNA-seq dataset will be crucial for the identification of chromatin and transcriptional regulators that mediate EC-subtype-specific responses to metabolic disease.

The EC-pneumocyte population was significantly reduced in the lungs with obesity (Fig. 6d). A similar loss of this population was reported in mouse models of pulmonary arterial hypertension^55^, suggesting a general loss of this population in diseased states. As the EC-pneumocyte population has only been detected via scRNA-seq analysis, we confirmed the presence of the EC-pneumocytes in mouse lungs via FISH (Fig. 3h). EC-pneumocytes express high levels of genes related to surfactant production (*Sftpa1*, *Sftpb*, *Sftpb* and *Sfta*), which are important in innate defense against pathogens and are typically expressed by pneumocyte type II cells in the lungs^56^. Moreover, our data revealed that immune response-related genes, including *Hc* (hemolytic complement), *Ptprf* and *Cxcl15*, and lysosomal genes, including *Lyz1* and *Lyz2*, were strongly enriched in EC-pneumocytes relative to other lung EC clusters. Thus, it is tempting to speculate that the loss of this population in obesity leads to reduced immunity against respiratory infections, such as influenza and severe acute respiratory syndrome coronavirus 2.

Platelet activation and adhesion is related to tissue and EC damage^39^. We uncovered that obesity is associated with increased platelet activation and accumulation in the liver endothelium, which progresses with sustained obesity (Fig. 5l). Platelet activation is an important defense mechanism that leads to increased inflammation and immune cell recruitment^57^. Metabolic syndrome and insulin-resistant obese individuals show elevated platelet counts and P-selectin^58,59^, which is consistent with our findings. Antiplatelet treatment results in reduced platelet–endothelial adhesion and has shown promising results as a therapy for non-alcoholic steatohepatitis^60^. As activation of ECs is instrumental for platelet aggregation, targeting endothelial adhesion molecules to reduce platelet-orchestrated inflammation in metabolic and chronic liver disease may be a new alternative therapeutic strategy.

Guided by our scRNA-seq data, we undertook tracing studies with fluorescent dextran dyes and uncovered reduced transport across fenestrated ECs in the choroid plexus in brains of obese animals (Fig. 3t). The choroid plexus is an important metabolic and secretory compartment of the brain that produces cerebrospinal fluid to maintain a homeostatic neural environment^61^. Alterations in choroid plexus morphology and function are associated with aging and Alzheimer’s disease^33,34^. Reduced transport across the choroid plexus is observed in individuals with Alzheimer’s disease^62^, while reduced permeability of the choroid plexus has been reported in animal models of inflammatory bowel disease, where it is associated with increased anxiety and memory deficiency^32^. Taken together with our transcriptomic and tracer data, it is possible that choroid plexus dysfunction may be one mechanism that causes deregulation of neural homeostasis in the context of obesity.

Obesity is a risk factor for chronic kidney disease and glomerulopathy development^63,64^. Obesity leads to increased sodium reabsorption, hypertension and glomerular hyperfiltration, which are associated with dysfunction of tubular and glomerular epithelial cells^65^. Here, we show that specialized kidney EC populations, such as mECs and gECs, are strongly impacted by obesity and show unique transcriptional changes (Fig. 3l–o and Extended Data Fig. 7e–l). Furthermore, dietary intervention was unable to restore the majority of obesity-induced transcriptional changes in kidney ECs, such as the upregulation of metabolic genes in mECs (Fig. 6i). This likely reflects the low regenerative capacity of the kidneys versus the higher regenerative capacity of organs such as the liver^66–68^, where ECs show improved transcriptional profiles on a reversion diet (Fig. 4f). In kidney gECs, we observed DLK1, which encodes an inhibitor of Notch signaling^30^, to be strongly upregulated in obesity (Fig. 3o). As DLK1 is thought to inhibit inflammation in the kidney^69^, the upregulation of DLK1 may be a protective response to obesity-induced damage. To date, major treatments for diabetes- and obesity-associated kidney dysfunction have focused on the renin–angiotensin–aldosterone axis and the regulation of vascular tone^70^. By uncovering unique transcriptional networks deregulated in renal EC subtypes in obesity, we provide here new directions to explore for therapeutics for treating kidney dysfunction in metabolic disease.

The increase in lipids and AT expansion in obesity creates a necessity for endothelial vascularization^71^. We observed an increase in EC angiogenesis, proliferation and ECM remodeling following a 3-month WD (Fig. 2e–j), consistent with a ‘healthy’ compensatory mechanism for fat expansion. However, sustained obesity resulted in reduced angiogenesis, particularly in the subcutaneous AT (Fig. 5a), along with an induction of *Hif1a* expression in visceral AT ECs (Extended Data Fig. 10a). These changes in sustained obesity are likely associated with increased hypoxia, which promotes inflammation and accumulation of immune cells in AT, leading to AT dysfunction^72,73^. Furthermore, the upregulation of ECM genes was less pronounced with sustained obesity (Fig. 5c and Extended Data Fig. 10d). As loss of angiogenic potential and improper extracellular remodeling are key characteristics of adipose dysfunction^36^, targeting angiogenesis, vascularization and EC function with therapeutics is likely to improve AT health in the context of obesity.

In conclusion, our work catalogs obesity-induced changes in the endothelium and provides the foundation for a better understanding of vascular dysfunction in metabolic disease and obesity-associated comorbidities. The molecular networks we have uncovered are candidate tissue-specific therapeutic target pathways to ameliorate EC dysfunction in a wide variety of disorders.

## Methods

### Animal models

All experiments were performed in accordance with the animal ethics laws of Saxony, Germany, and were approved by the state animal ethics committee (Landesdirektion Sachsen, Leipzig, Germany). Male C57BL/6N mice were put on a WD (Ssniff Spezialdiäten, D12331) or control chow diet (Ssniff Spezialdiäten, V1534) starting at ~8 weeks of age. Animals were maintained on the respective diets for 3 months and weighed weekly. For the ‘reversion’ experiments, animals maintained on a WD for 3 months were changed to a chow diet and analyzed 1 or 3 months later. Body composition was measured using the Minispec BCA Analyzer LF110 (Bruker Biospin). Mice were maintained on a 12-h day/12-h night cycle. Water and food were provided ad libitum. The temperature was maintained at 22 ± 2 °C, with humidity maintained at 55 ± 10%.

### Statistical analysis

Data, which were not derived from scRNA-seq data analyses, are provided as mean ± s.e.m. All statistical tests were exclusively undertaken on biological replicates. Comparison of data between the indicated two groups was performed using a two-sided Student’s *t*-test. A *P* value of <0.05 was considered statistically significant.

### Preparation of single-cell suspensions

#### Brain

Brains were dissected and rinsed in ice-cold PBS. The olfactory bulb and cerebellum were removed. The brain was then dissociated with the Neural Dissociation kit P (Miltenyi Biotec, 130-092-628), as per the manufacturer’s instructions, via the gentleMACS Octo Dissociator system with heaters (MACS Technology, Miltenyi Biotec) using the 37C_NTDK_1 program. Cells were transferred via a 20-gauge syringe through a 70-µm cell strainer and into a 50-ml Falcon tube. Cells were collected by centrifugation (4 °C, 300*g*, 5 min).

#### Lungs

The lungs were surgically removed, rinsed in ice-cold PBS and transferred into a gentleMACS C tube (Miltenyi Biotec, 130-096-334) containing tissue digestion buffer (TDB). TDB consisted of 1× penicillin/streptomycin (Thermo Fisher Scientific, 15140122), 2× antibiotic–antimycotic (Thermo Fisher Scientific, 15240062), 1 mM sodium pyruvate (Thermo Fisher Scientific, 1360070), 1× MEM non-essential amino acids solution (Thermo Fisher Scientific, 11140035), 0.13 Wunsch units (WU) Liberase (Merck, 5401127001) and 160 U DNase I (Sigma-Aldrich, D4527-10KU) made up in KnockOut DMEM (Thermo Fisher Scientific, 10829018). Each sample was further dissociated with the gentleMACS Octo Dissociator system with heaters using the 37C_m_LDK_1 protocol. The cell suspension was filtered through a 70-µm cell strainer, and the cell strainer was rinsed once with 10 ml of wash buffer (WB; containing 0.5% bovine serum albumin (BSA; BSA Fraction V, Sigma-Aldrich, 10735096001) and 2 mM EDTA (Thermo Fisher Scientific, 14190-094) in PBS). Cells were collected via centrifugation (4 °C, 300*g*, 5 min).

#### Heart

The heart was surgically removed, rinsed in ice-cold PBS, cut into approximately 10 pieces and transferred into a gentleMACS C tube containing TDB supplemented with 0.13 WU Liberase (Merck, 5401127001) and 80 U DNase I (Sigma-Aldrich, D4527-10KU). Each sample was dissociated with the gentleMACS Octo Dissociator system with heaters using the preprogrammed protocol 37C_NTDK_1. The cell suspension was transferred using a 20-gauge needle and filtered through a 70-µm cell strainer, rinsed (10 ml of WB), collected by centrifugation and used for staining.

#### Kidneys

The kidneys were surgically removed and rinsed in ice-cold PBS. Kidneys were cut into small pieces using surgical scissors and transferred into a gentleMACS C tube containing TDB supplemented with 0.13 WU Liberase (Merck, 5401127001) and 80 U DNase I (Sigma-Aldrich, D4527-10KU). Each sample was dissociated with the gentleMACS Octo Dissociator system with heaters using the preprogrammed protocol 37C_Multi_E. The cell suspension was filtered through a 70-µm cell strainer. The gentleMACS C tube and the cell strainer were subsequently rinsed in 10 ml of WB. Cells were collected via centrifugation (4 °C, 300*g*, 5 min), washed once in 10 ml of WB and used for staining.

#### Liver

The liver was dissected, rinsed in ice-cold PBS, minced using scissors and transferred into a gentleMACS C tube containing TDB supplemented with 0.13 WU Liberase (Merck, 5401127001) and 80 U DNase I (Sigma-Aldrich, D4527-10KU). Each sample was dissociated with the gentleMACS Octo Dissociator system with heaters using the preprogrammed protocol 37C_m_LIDK_1. The cell suspension was filtered through a 70-µm cell strainer and further rinsed with 10 ml of WB. Cells were collected by centrifugation (4 °C, 300*g*, 5 min) and used for staining.

#### AT

The visceral and subcutaneous AT were surgically removed, rinsed in ice-cold PBS and transferred into a gentleMACS C tube containing adipose digestion buffer (DMEM (Gibco, 41966-029), 1% penicillin/streptomycin (Thermo Fisher Scientific, 15140122) and 0.2% collagenase II (Gibco, 17101-015)). Using surgical scissors, the AT was cut into small pieces. Samples were then dissociated with the gentleMACS Octo Dissociator using the preprogrammed protocol 37C_mr_ATDK_1. The cell suspension was filtered through a 300-µm cell strainer. The gentleMACS C tube and the cell strainer were subsequently rinsed twice with 10 ml of WB. Cells were collected by centrifugation (4 °C, 300*g*, 5 min). The adipose layer at the top was discarded, the cell pellet was washed once in 10 ml WB and the cells were subsequently used for staining.

### EC staining and isolation by FACS

For the 3-month timepoint (WD 3 months and chow 3 months), cell suspensions from the liver and kidneys were treated with red blood cell lysis buffer (0.154 M NH_4_Cl, 0.01 M KHCO_3_ and 0.1 mM EDTA for 3 min at room temperature), washed once in WB and subsequently used for staining. Myelin was removed from brain samples using myelin removal beads (Miltenyi Biotec, 130-096-733), as per the manufacturer’s instructions, before staining. Similarly, ECs were removed from lung suspensions using CD326 (EpCAM) microbeads (Miltenyi Biotec, 130-105-958) according to the manufacturer’s instructions.

For the 4-month (WD 4 months, chow 4 months and reversion 1 month) and 6-month (WD 6 months, chow 6 months and reversion 3 months) timepoints, cell suspensions from the liver, heart, kidneys, brain and lungs were treated with CD31 MicroBeads (Miltenyi Biotec, 130-097-418) to enrich for ECs before staining. CD31 enrichment was performed according to the manufacturer’s instructions.

FACS isolation of ECs was performed as previously described^74,75^. Briefly, all samples were stained with CD45-PE (BD Pharmigen, 533081; 1:400) and CD31-APC (eBioscience, 17-0311-85; 1:250) antibodies diluted in FACS buffer (2% fetal calf serum in PBS). Staining was done on ice in a total volume of 200 µl. Cells were washed in 14 ml of FACS buffer, collected by centrifugation (4 °C, 300*g*, 5 min), resuspended in FACS buffer containing 1 µg ml^–1^ propidium iodide and passed through a 100-µm cell strainer into a FACS tube. Cells were sorted on a FACS Melody or FACS Aria (BD Biosciences). Single cells were selected based on forward and side scatter. Dead cells were removed using propidium iodide. ECs were gated based on CD31^+^ and CD45^low^ expression.

### Single-cell workflow

scRNA-seq was performed using a 10x Next GEM Single-Cell 3′ GEM kit v3.1 (10x Genomics) according to the manufacturer’s protocol. Briefly, an equal number of FACS-isolated CD31^+^CD45^low^ cells from three biological replicates per condition were pooled, centrifuged (4 °C, 300*g*, 5 min), resuspended at ~1,000 cells per µl and immediately loaded into the 10x Chromium controller. Separate 10x Genomics reactions were used for each organ, timepoint and condition, with ECs pooled from three mice per group. Generated libraries were sequenced on an Illumina NovaSeq with >27.5 × 10^3^ reads per cell followed by demultiplexing and mapping to the mouse genome (build mm10) using CellRanger v5.0 (10x Genomics).

### Bioinformatics analyses

#### Data preprocessing

Gene expression matrices were generated using the CellRanger software v5.0.1 (10x Genomics) with standard settings and mapping to the mm10 reference mouse genome. The following data analysis was performed using Seurat package v3.0 (ref. ^76^). First, we performed data filtering steps by removing low expressed genes and low-quality cells from further analyses: (1) genes with 0 raw counts were removed, (2) cells with <500 or >6,000 uniquely expressed genes or with >25,000 unique molecular identifiers were excluded and (3) cells with a high percentage of mitochondrial genes (>20%) were removed. The data were normalized using the NormalizeData() function, the 3,000 most variable features were detected with FindVariableFeatures(), data were scaled with ScaleData() and the first 30 principal components were calculated with RunPCA() and used for clustering (RunUMAP(), FindNeighbours() and FindClusters()). Doublets were removed with the DoubletFinder package according to the doublet rates provided by 10x Genomics. Second, we merged the data from all organs into one object and annotated major cell types. Clusters were annotated based on the expression of the following markers: *Pecam1* (*Cd31*) and *Cdh5* (vascular ECs); *Prox1* and *Lyve1* (LECs); *Dcn*, *Pdgfra* and *Col1a1* (fibroblasts); *Myh11*, *Acta2* and *Tagln* (smooth muscle cells); *Pdgfrb*, *Cspg4* and *Anpep* (pericytes) and *Ptprc*, *Igkc* and *Cd52* (hematopoetic cells). Non-ECs, which showed no *Pecam1* or *Cdh5* expression, were removed from all downstream analyses. For further analyses, cells were separated according to the organ of origin for subclustering and differential gene expression. DEGs were obtained using the FindMarkers() function based on a Wilcoxon rank-sum test with Benjamini–Hochberg *P* value correction. Significant DEGs were identified by the criteria Benjamini–Hochberg-adjusted *P* value of <0.05 and | log (FC) | > 0.1. Differential expression is expressed on a natural log (log_e_) scale.

#### Heat maps

Heat maps were generated using log (FC) of DEGs or *z* scores calculated for average expression per cluster, as indicated in the figure legends, via the ggplot2 package. For better visual representation, upper and lower value cutoffs were introduced as stated on the heat maps. For reversion experiments (Figs. 4–6), data for the WD and reversion groups at each timepoint were standardized to the associated chow controls at the same timepoint.

#### Designation of art, cap and ven ECs

General EC subtypes were assigned based on marker genes from previous studies^11,16,18^. As such, art ECs in general were assigned by expression of *Fbln5*, *Gkn3*, *Hey1* and *Mgp*, with additional markers *Aqp1* (kidney) and *Plac8* and *Adgrg6* (liver). Cap ECs were assigned based on expression of *Car4* and *Rgcc* as well as *Sema3c* (lungs), *Plpp3* (kidneys) and *Stab2* and *Lyve1* (liver). Ven ECs were assigned based on the expression of *Vcam1* and *Vwf*, whereas *Nr2f2* and *Lcn2* were used for brain, *Igf1* for kidneys and *Selp* and *Bmp4* for liver.

#### Designation of cell clusters

Every organ was analyzed separately for subclustering and differential gene expression analysis. Clusters were assigned based on the art–cap–ven markers and other organ-specific markers using the FindAllMarkers() function. Proliferating and angiogenic populations were defined by the expression of *Mki67* (ref. ^77^), *Top2a*^78,79^ and *Apln*, *Col4a2* and *Trp53i11*, respectively. EC-Hb cells were defined by *Hba-a1* and *Hbb-bs*, and EC-AP1 cells were defined by enrichment of *Junb*, *Fos* and *Fosb*. LECs were defined by the expression of *Flt4*, *Ccl21a*, *Lyve1* and *Fgl2*. For analyses of later timepoints and reversion at 4 and 6 months, cells were incorporated with the original object for each organ using the merge() function and reclustered, and cluster identities were assigned based on the original 3-month timepoint. For the quantification of platelet-positive ECs, we applied the following criteria: >1 count for any of the platelet-specific genes *Pf4*, *Ppbp* and *Nrgn*.

#### Correlation analysis of DEGs

A list of genes that showed an increase or decrease of log (FC) > 0.1 in the obese versus chow conditions in the indicated EC subpopulations was generated. Pairwise correlation analysis was then performed on genes that were expressed in both datasets being compared. Correlation coefficients representing Pearson’s *r* values were calculated.

#### Identification of genes most consistently changing across organs

For the indicated timepoints, genes were selected that were detected in all organs. The log (FC) values in the obese versus control conditions were then compiled in a matrix for each gene across all organs. Genes were ranked by average log (FC) across all seven organs. The top genes up- and downregulated, showing a minimum average | log (FC) | > 0.2, were included.

#### GO and pathway enrichment analysis

GO and pathway enrichment analyses were performed using Enrichr^80^. Selected pathways and GO terms significantly enriched with a Benjamini–Hochberg-adjusted *P* value of <0.05 are presented. Data are presented on a –log_10_ scale.

#### Transcription factor binding motif analysis

To identify putative transcriptional regulators of *Fabp1*, genomic sequences of the *Fabp1*, *Fabp4*, *Fabp5*, *Cd36* and *Abca1* promoter regions (1,000 kilobases upstream of the transcription start site) were extracted from the UCSC Table Browser^81^ using the mm10 reference genome. Promoter regions were analyzed with AME online tools v5.4.1 (ref. ^82^) via the HOMOCOMO v11 motif database. Top enriched motifs for the mouse were subsequently mapped to the *Fabp1* promoter using the CentriMo online tool v5.4.1 (ref. ^82^).

#### Integration of GWAS data

GWAS data were searched and downloaded from the NHGRI-EBI Catalog of human GWASs^40^. This database contains all human GWASs that meet the NHGRI-EBI quality criteria (more than 100,000 single-nucleotide polymorphisms (SNPs) analyzed in study). Human genetic variants showing statistically significant association (*P* < 10^−5^) with the indicated disease were compared to DEGs in our scRNA-seq dataset. Child trait data were excluded. For dot plots comparing GWAS SNP-associated genes and DEGs in our dataset, we used the log (FC) of WD versus chow cohorts at the 6-month timepoint and plotted it against the –log_10_ (*P* value) of the disease-associated SNP of that gene.

### Metabolomics

Metabolomics analyses were performed using gas chromatography–mass spectrometry (GC–MS) as previously described^75^. Briefly, serum samples were collected from animals at the time of death. A total of 25 µl of serum was mixed with 200 µl of 100% ice-cold methanol. Norvaline and D27-myristic acid were used as spike-in controls. The samples were frozen for at least 24 h at –80 °C. The mixture was centrifuged (20,000*g*, 4 °C, 5 min), and the supernatant was kept for analysis. For measurement, the supernatant was dried in a speed-vac (room temperature), and samples were resuspended in 10 µl of pyridine with 10 mg ml^−1^ methoxyamine and incubated for 1 h at 30 °C. Samples were centrifuged (20,000*g*, 3 min), and the supernatant (7.5 µl) was transferred to GC–MS tubes. Subsequently, metabolites were derivatized by the addition of 15 µl of *N*-(*tert*-butyldimethylsilyl)-*N*-methyl-trifluoroacetamid with 1% *tert*-butyldimethylchlorosilane (Sigma-Aldrich, 375934) and incubation for 60 min at 80 °C. Metabolites were measured using a DB5-MS GC column in a 7890 GC system (Agilent Technologies) combined with a 5977 MS system (Agilent Technologies).

### Immunofluorescence

Obese and chow animals were killed and intracardially perfused with 15 ml of PBS (Sigma, D8537) and 15 ml of freshly prepared paraformaldehyde, pH 7.4 (Sigma, P6148). Tissues were then processed as described below.

#### Liver

The liver was cut into approximately 0.5-cm^3^ pieces and fixed with 4% formaldehyde (overnight at 4 °C). Tissues were embedded in paraffin, and 5-µm sections were cut using a microtome (Thermo Scientific, HM355S) and attached to Superfrost slides (Epredia, J1800AMNZ).

Immunostaining was performed using antibodies for CD62P (P-selectin, Psel.KO.2.7; Novus Biologicals, NB100-65392; 1:100) and CD31 (Abcam, ab28364; 1:50). Sections were deparaffinized and hydrated followed by postfixation in ice-cold acetone for 1 min and washing in PBS for 10 min. Antigen retrieval was performed using antigen-unmasking solution (Vector). Sections were incubated for 1 h in blocking solution (1.5% fetal calf serum and 3% BSA prepared in PBS), followed by the addition of primary antibodies (CD62P and PECAM1) overnight at 4 °C. Following washing in PBS for 10 min, sections were incubated for 120 min with corresponding secondary antibodies. Sections incubated with only secondary antibodies were used as negative controls. Sections were then washed twice for 10 min each in PBS and mounted in Vectashield containing DAPI and visualized using a fluorescence microscope (Leica). Image exposure and acquisition settings were set using negative controls (without primary antibodies), and similar settings were used for all sections. Quantification was done using ImageJ software via the colocalization color map plug-in, and index of correlation (*I*_corr_) score was plotted.

#### Visceral and subcutaneous AT

The tissue was dissected and fixed with 40 ml of 4% paraformaldehyde (overnight at 4 °C) and then sequentially incubated in 40 ml of 10%, 20% and 30% sucrose for 24 h each. Pieces around 1 cm^3^ were cut, and tissue was embedded in OCT (Cell Path, KMA-0100-00A), frozen on dry ice and stored at –80 °C. Sections of 40 μm were generated using a Cryostat (Leica, CM1950), and sections were attached to Superfrost slides (Epredia, J1800AMNZ).

Immunostaining was performed on cryosections. Slices were incubated in PBS for 30 min and permeabilized in 0.5% Triton X-100 (Roth, 3051.3) in PBS for 30 min. Sections were washed three times for 5 min each in PBS and blocked in blocking solution (5% BSA (PanReac AppliChem, A1391), 10% glycine (Roth, 0079.3) and 0.2% Triton X-100 (Roth, 3051.3)) for 2 h. Primary antibodies were incubated for 48 h at 4 °C (anti-CD31, 1:50 (Abcam, ab56229); anti-integrin-β1, 1:50 (sc-374429)) in blocking solution. Following three washes in PBS (5 min each), samples were incubated for 120 min with the corresponding secondary antibodies in blocking solution (Alexa Fluor 647 goat anti-rabbit IgG (H + L), Life Technologies, A21244; Alexa Fluor 488 donkey anti-rat IgG (H + L), Life Technologies, A21208; Alexa Fluor 555 goat anti-mouse IgG (H + L), Life Technologies A28180; 1:300). Sections were then washed twice for 2 min in 0.2% Triton X-100 in PBS, washed twice for 5 min with PBS and mounted with Mowiol (Roth, 0713.2) containing DAPI (Roth, 6335.1) and visualized using a confocal microscope (Zeiss, LSM980). The same image exposure and acquisition settings were used for all sections. Quantification was done using ImageJ software. Four regions per sample (*n* = 3 animals per dietary group) were acquired. Expression of integrin-β1 was standardized to the CD31 signal. Normal distribution of data was evaluated with a Shapiro test (R studio), and an unpaired *t*-test was performed (GraphPad Prism 8.4.3).

#### Kidneys

Five-micron-thick paraffin sections were prepared. Sections were deparaffinized with xylene and rehydrated with graded ethanol washes. Sections were then cooked in Tris-based antigen-unmasking solution to retrieve the epitopes. Following antigen retrieval, sections were covered with TrueBlack (Biotium, 23007) for 1 min and washed twice with PBS for 10 min. Blocking was done at room temperature for 1 h in 3% donkey serum and M.O.M. blocking reagent (Vector labs). Slides were incubated with primary antibodies raised against DLK1 (1:200; Abcam, ab119930) and CD31 (1:200; Abcam, ab28364) for 48 h at 4 °C. Slides were washed three times with PBS and incubated with corresponding secondary antibodies (anti-mouse Alexa Fluor 546, A10036; anti-rabbit Alexa Fluor 488, SA5-10038; 1:200) for 2 h followed by three washes in PBS. Sections were mounted using VECTASHIELD mounting medium containing DAPI (Vectashield plus Antifade DAPI, Vector lab, H-2000). Images were acquired using a Leica Thunder microscope. The exposure settings and laser gain were kept constant for each condition, and analysis was performed using NIH ImageJ software.

### Choroid plexus barrier permeability/dye uptake assay

The following protocol was used with minor modifications^83^. Obese and chow-fed mice were intraperitoneally administered 100 µl of tracer solution (2 mM dextran fluorescein, 3 kDa (Thermo Fisher, D3306), and 2 mM dextran Texas Red, 70 kDa (Thermo Fisher, D1864)). Mice injected with PBS were used as a negative control. Five minutes after tracers were injected, animals were slowly anesthetized in an isoflurane chamber. Ten minutes after (that is, 15 min after dye injection), a cardiac puncture was performed, and 500 µl of blood was collected from each animal. Mice were intracardially perfused with 15 ml of PBS (Sigma, D8537), the brain was isolated, and the two hemispheres were separated. One hemisphere was embedded in OCT (Cell Path, KMA-0100-00A), frozen on dry ice and preserved at –80 °C. Blood samples were centrifuged at 10,000*g* for 10 min at 4 °C, and serum was collected. Fifty microliters of diluted serum (2:5 in PBS) was used to perform fluorescence measurements at excitation/emission of 595/615 and 494/521 nm via a multimode microplate reader FlexStation 3 (Molecular Devices). The fluorescence intensity from serum was used in the next step as a control.

Sections (10 µm) were generated using a cryostat (Leica, CM1950) and attached to Superfrost slides. Cryosections were fixed with freshly prepared 4% paraformaldehyde, pH 7.4 (Sigma, P6148) for 30 min, permeabilized with 0.2% Triton X-100 in PBS for 15 min and blocked in a solution composed of 5% BSA, 10% glycine and 0.2% Triton X-100 in PBS for 1 h. Incubation with anti-CD31 (Abcam, ab28364; 1:50) was performed overnight in blocking solution at 4 °C. After three washes in PBS (5 min each), sections were incubated for 120 min with the corresponding secondary antibody. Sections were then washed twice for 2 min each in 0.2% Triton X-100 and for 5 min in PBS and mounted in Mowiol (Roth, 0713.2) containing DAPI and visualized using a confocal microscope (Zeiss, LSM980). The same image exposure and acquisition settings were used for all sections. Quantification was done using ImageJ software. Four regions of the choroid plexus (*n* = 5 mice per dietary group) were acquired, and raw fluorescence (RFU) values were normalized against raw fluorescence measurements of the serum from the plate reader. Normal distribution of data was evaluated with a Shapiro test (R studio), and an unpaired *t*-test was performed (GraphPad Prism 8.4.3).

### FISH

Obese and chow animals were killed and intracardially perfused with 15 ml of PBS followed by 15 ml of freshly prepared 4% paraformaldehyde, pH 7.4 (Sigma, P6148). The lungs were promptly dissected and further fixed in 4% paraformaldehyde overnight at 4 °C.

Tissues were cryoprotected and processed as previously described^84^. Lungs were placed in OCT (Cell Path, KMA-0100-00A), frozen on dry ice and preserved at –80 °C. Seven-micron sections were cut using a Cryostat (Leica, CM1950) and attached to Superfrost slides, which had previously been treated with poly-l-lysine^84^. Fixation, permeabilization, proteinase K digestion (1:1,500), hybridization and mounting were performed as previously described^84^. The fluorescent probes were ordered from Biosearch Technologies and possessed between 30 and 48 unique hybridization primers per target gene (Supplementary Table 8). *Pecam1* probes were labeled with Fluor Red 590, and all other probes were labeled with Quasar 670 to allow for multiplexing. *Tubb3* was used as a negative control, as it is typically not expressed in healthy lungs. Sections were visualized using a confocal microscope (Zeiss, LSM980). Four regions per sample (*n* = 3 animals per dietary group) were acquired.

### Primary mouse EC culture

Liver and lungs were dissected from female C57/BL6N mice at 6 to 9 weeks old and placed in ice-cold sterile PBS. Samples were moved to a sterile tissue culture hood, and single-cell suspensions were prepared using the Miltenyi Octo Dissociator as described above. ECs were enriched from the single-cell suspensions using CD31 MicroBeads (Miltenyi Biotec, 130-097-418), following the manufacturer’s instructions, under sterile conditions. Cells were cultured in collagen-coated (Sigma, C8919) plates with EC growth medium (Cell Applications, 211-500).

### Fatty acid, glucose and insulin treatment

#### Fatty acid conjugation

A fatty acid cocktail was prepared with the following fatty acids purchased from Sigma and dissolved in ethanol: 1 mM c14:0 (myristic acid, M3128), 26.7 mM c16:0 (palmitic acid, P0500), 8 mM c18:0 (stearic acid, S4751), 36.6 mM c18:2 (linoleic acid; L1376), 21 mM c18:1 (oleic acid; E4637) and 6.4 mM c20:4 (arachidonic acid, Merck, 181198). These are six of the most common free fatty acids found in human serum. Our chosen combination of fatty acids represents the relative quantities of these six fatty acids in human serum^23,85^. The fatty acid cocktail (100 mM) was conjugated with 10% BSA (Sigma, A8806) for 2 h with shaking at 1,500 r.p.m. at 37 °C in a thermoshaker.

#### EC treatment

Liver ECs were treated with metabolic stressors 24 h after initial culture, while lung ECs were treated at passage 3. Primary ECs were treated with 30 mM glucose (Gibco, A24940-01), 1:1,000 insulin (~10 µg ml^–1^ final concentration; Sigma, I9278) and the fatty acid cocktail at three concentrations (100, 400 and 800 µM) for 24 h. These three concentrations were used because the normal concentration of free fatty acids in human serum is ~450 μM (ref. ^23^), while it is increased around twofold in obesity^24^. RNA was subsequently isolated for quantitative PCR with reverse transcription (qRT–PCR) analysis. For rescue experiments, ECs were treated with 5 µM GW6471 (Sigma, G5045) or 1 µM MRT67307 (Sigma, SML0702) for 1 h, followed by a 23-h treatment with 800 µM fatty acid cocktail and the respective inhibitor together. After treatment, ECs were washed twice with ice-cold PBS, and RNA was isolated using an RNeasy Mini kit (Qiagen, 74106) according to the manufacturer’s instructions.

#### cDNA synthesis and quantitative PCR analysis Isolation of cardiac ECs

CD31^+^CD45^low^ cells were isolated by FACS as described above, and RNA was isolated using a Qiagen RNeasy kit.

#### Isolation of lung ECs

CD31^+^CD45^low^ cells were isolated by FACS as described above, and RNA was isolated using a Qiagen RNeasy kit.

Following DNase digestion, RNA was reverse transcribed to cDNA using a Maxima first-strand cDNA synthesis kit (Thermo Fisher Scientific, K1671). For each experiment, an equal quantity of input RNA was used for each sample for the cDNA reaction. qRT–PCR reactions were run in triplicate with Power SYBR Green PCR master mix (Life Technologies, 4368708) on an LC480 instrument (Roche). The relative expression changes were normalized to *Rplp0*. For heart EC samples, the *Rplp0*-set 2 primers were used. The full list of primers is provided in Supplementary Table 9.

### Reporting summary

Further information on research design is available in the Nature Portfolio Reporting Summary linked to this article.

### Supplementary information

Reporting Summary

Supplementary Table 1 Supplementary Tables 1–9.

### Source data

Source Data Fig. 1 Statistical source data.

Source Data Fig. 2 Statistical source data.

Source Data Fig. 3 Statistical source data.

Source Data Fig. 4 Statistical source data.

Source Data Fig. 5 Statistical source data.

Source Data Fig. 6 Statistical source data.

Source Data Fig. 7 Statistical source data.

Source Data Extended Data Fig. 1 Statistical source data.

Source Data Extended Data Fig. 2 Statistical source data.

Source Data Extended Data Fig. 3 Statistical source data.

Source Data Extended Data Fig. 4 Statistical source data.

Source Data Extended Data Fig. 5 Statistical source data.

Source Data Extended Data Fig. 6 Statistical source data.

Source Data Extended Data Fig. 7 Statistical source data.

Source Data Extended Data Fig. 8 Statistical source data.

Source Data Extended Data Fig. 9 Statistical source data.

Source Data Extended Data Fig. 10 Statistical source data.

## Data Availability

All gene expression data are provided on the website https://obesity-ecatlas.helmholtz-muenchen.de. Processed data can be interrogated using the graphical user interface provided, and normalized count matrices can be downloaded from the website by clicking the ‘download 5had’ button under each dataset. Differential expression analysis between any two populations of interest can also be performed via the website. Source data are provided with this paper.

## Code availability

The code used for bioinformatics analysis can be accessed via GitHub at https://github.com/Osynchronika/sc_EC_obesity_atlas.
