# Supplementary material for: Single-cell profiling of vascular endothelial cells reveals progressive organ-specific vulnerabilities during obesity
Source: Nat Metab. 2022 Nov 18;4(11):1591–610. doi: 10.1038/s42255-022-00674-x (PMC9684070; doi:10.1038/s42255-022-00674-x)
Supplement: Supplementary file 1 — Reporting Summary [file 42255_2022_674_MOESM1_ESM.pdf]

nature portfolio

Corresponding author(s): Bilal Sheikh

Last updated by author(s): Sep 21, 2022

Reporting Summary

Nature Portfolio wishes to improve the reproducibility of the work that we publish. This form provides structure for consistency and transparency in reporting. For further information on Nature Portfolio policies, see our Editorial Policies and the Editorial Policy Checklist.

Statistics

For all statistical analyses, confirm that the following items are present in the figure legend, table legend, main text, or Methods section.

n/a	Confirmed	
☐	☒	The exact sample size (*n*) for each experimental group/condition, given as a discrete number and unit of measurement
☐	☒	A statement on whether measurements were taken from distinct samples or whether the same sample was measured repeatedly
☐	☒	The statistical test(s) used AND whether they are one- or two-sided<br>*Only common tests should be described solely by name; describe more complex techniques in the Methods section.*
☒	☐	A description of all covariates tested
☐	☒	A description of any assumptions or corrections, such as tests of normality and adjustment for multiple comparisons
☐	☒	A full description of the statistical parameters including central tendency (e.g. means) or other basic estimates (e.g. regression coefficient) AND variation (e.g. standard deviation) or associated estimates of uncertainty (e.g. confidence intervals)
☐	☒	For null hypothesis testing, the test statistic (e.g. *F*, *t*, *r*) with confidence intervals, effect sizes, degrees of freedom and *P* value noted<br>*Give P values as exact values whenever suitable.*
☒	☐	For Bayesian analysis, information on the choice of priors and Markov chain Monte Carlo settings
☒	☐	For hierarchical and complex designs, identification of the appropriate level for tests and full reporting of outcomes
☐	☒	Estimates of effect sizes (e.g. Cohen's *d*, Pearson's *r*), indicating how they were calculated

Our web collection on statistics for biologists contains articles on many of the points above.

Software and code

Policy information about availability of computer code

Data collection	Single cell RNA-seq data was collected from FACS sorted CD31 high, CD45 low cells from male mice using the 10x genomics platform. The full methods are enclosed in this document below.
Data analysis	Gene expression matrices were generated using the CellRanger software v5.0.1 (10x Genomics). Data analysis was performed using Seurat package v3.0. Data were normalized using the NormalizeData() function, the 3000 most variable features detected with FindVariableFeatures (), data scaled with ScaleData(), the first 30 principal components calculated with RunPCA(), and used for clustering (RunUMAP(), FindNeighbours(), FindClusters()). Doublets were removed with the DoubletFinder package according to the doublet rates provided by 10x Genomics. Differentially expressed genes were obtained using the FindMarkers() function based on a Wilcoxon Rank Sum test with Benjamini p-value correction.<br>Heatmaps were generated using the ggplot2 package.<br>Gene ontology and pathway enrichment analyses were performed using Enrichr (v. Mar2021).<br>TF binding motif analysis was performed using AME online tools v5.4.1 via the HOMOCOMO v11 motif database.<br>The code used for bioinformatics analysis can be accessed via GitHub:<br>https://github.com/Osynchronika/sc_EC_obesity_atlas

For manuscripts utilizing custom algorithms or software that are central to the research but not yet described in published literature, software must be made available to editors and reviewers. We strongly encourage code deposition in a community repository (e.g. GitHub). See the Nature Portfolio guidelines for submitting code & software for further information.

Data

Policy information about availability of data

All manuscripts must include a data availability statement. This statement should provide the following information, where applicable:

- Accession codes, unique identifiers, or web links for publicly available datasets
- A description of any restrictions on data availability
- For clinical datasets or third party data, please ensure that the statement adheres to our policy

All data are provided on the website:
https://obesity-ecatlas.helmholtz-muenchen.de
Processed data can be interrogated using the graphical user interface provided and normalized count matrices downloaded from the website by clicking the "download 5had" button under each dataset. Differential expression analysis between any two populations of interest can also be undertaken via the website.
The code used for bioinformatics analysis can be accessed via GitHub:
https://github.com/Osynchronika/sc_EC_obesity_atlas
The NHGRI-EBI GWAS catalog can be accessed at: https://www.ebi.ac.uk/gwas/

Field-specific reporting

Please select the one below that is the best fit for your research. If you are not sure, read the appropriate sections before making your selection.

[x] Life sciences   [ ] Behavioural & social sciences   [ ] Ecological, evolutionary & environmental sciences

For a reference copy of the document with all sections, see nature.com/documents/nr-reporting-summary-flat.pdf

Life sciences study design

All studies must disclose on these points even when the disclosure is negative.

Sample size	Single cell RNA-seq: for each time point and condition, an equal number of CD31+ CD45 low cells were extracted from at least 3 different male mice prior to single cell analysis.<br>For other experiments, at least 3 biological replicates per experimental group were performed. The exact number of replicates is indicated in the figure legends.<br>As per good scientific practice, a minimum of 3 biological replicates were undertaken for each experiment. Where extra samples / animals were available, more than 3 replicates were used. No statistical method was used to determine sample size.
Data exclusions	Only cells showing expression of endothelial markers (Cdh5, Pecam1) were included and non-endothelial cells excluded from the analysis. This was done firstly during FACS sorting of ECs, and secondly during pre-processing of scRNA-seq data by excluding Pecam1- and Cdh5-negative cells, as the study primarily focused on ECs.<br><br>For quality control of the sequencing data, we used commonly used, pre-determined metrics to filter cells.<br>Cells where less than 500 unique genes (low quality or dead cells), or more than 6000 uniquely expressed genes (likely doublets) were detected were removed; cells with >25000 UMIs were excluded (likely doublets); cells with high percentage of mitochondrial genes (more than 20% of all detected transcripts) were removed (low quality or dead cells); cells identified as doublets with DoubletFinder R package were removed.
Replication	- Multiple replicates - at least 3 animals - were used for each condition and time point. Biological replicates showed similar results, and data from each biological replicate has been indicated as a separate point in the figures.<br>- Data were compared over multiple time points to observe changes over progressive / more severe obesity in the single cell dataset.
Randomization	Prior to administering animals with a Western diet, animals were randomly assigned into the "chow" or "Western diet" groups.<br>For reversion experiments - switching animals from a Western diet to a chow diet, animals on a Western diet were randomly assigned into the sustained obesity or reversion groups.
Blinding	As obese animals are obvious, blinding was not possible.

Reporting for specific materials, systems and methods

We require information from authors about some types of materials, experimental systems and methods used in many studies. Here, indicate whether each material, system or method listed is relevant to your study. If you are not sure if a list item applies to your research, read the appropriate section before selecting a response.

Materials & experimental systems

n/a	Involved in the study
	☒ Antibodies
☒	☐ Eukaryotic cell lines
☒	☐ Palaeontology and archaeology
	☒ Animals and other organisms
☒	☐ Human research participants
☒	☐ Clinical data
☒	☐ Dual use research of concern

Methods

n/a	Involved in the study
☒	☐ ChIP-seq
	☒ Flow cytometry
☒	☐ MRI-based neuroimaging

Antibodies

Antibodies used

CD45-PE (BD Pharmigen 533081, 1:400)
CD31-APC (eBioscience 17-0311-85, 1:250)
CD31 (for immunofluoresence) (Abcam ab28364, 1:50)
CD31 (for immunofluoresence) (Abcam ab56229, 1:50)
CD62P (P-selectin, Psel.KO.2.7, Novus Biologicals NB100-65392, 1:100)
ITGB1 (sc-374429, 1:50)
DLK1 (Abcam ab119930, 1:200)
Secondary antibodies:
AlexaFluor-546 anti-mouse IgG, A10036 (1:200)
AlexaFluor-488 anti-rabbit IgG, SA5-10038 (1:200)
Alexa Fluor 647 goat anti-rabbit IgG (H+L), Life technologies A21244 (1:300)
Alexa Fluor 488 donkey anti-rat IgG (H+L), Life technologies A21208 (1:300)
Alexa Fluor 555 goat anti-mouse IgG (H+L), Life technologies A28180 (1:300)

Validation

CD31-APC, CD45-PE: We have previously utilized these antibodies to isolate endothelial cells and ensured endothelial identity by single cell RNA-seq and qPCR / RNA-seq analysis for endothelial markers (Sheikh et al. 2020, Nature Cell Biology, Sheikh et al. 2019 iScience). During this study, we also undertook single cell sequencing on CD31-positive cells. As expected, we specifically obtained endothelial cells.
The CD31 Abcam antibodies (for IF) were verified by ensuring vascular staining on test sections. Secondary antibody only controls did not show this staining. We observed the expected staining of blood vessels, which have a very distinct pattern in tissues. The manufacturer (Abcam) has also provided verifications of vascular stainings in multiple organs using both antibodies. According to the manufacturer, the ab28364 antibody has been cited in more than 1,600 studies, while the ab56299 has been cited in more than 75 studies.
The ITGB1 antibody was tested by the manufacturer for specificity using Western blot on 3 different cell types. Expected band sizes between 100 and 130 kDa were observed. The antibody has been used in 60 studies (SantaCruz website). Knockout of ITGB1 leads to a loss of signal with this antibody suggesting that this antibody is specific (Lu et al. 2020, American Journal of Transplantation).
CD62P (P-selectin) antibody was verified by staining of activated platelets. It co-stained with CD42b, another marker of platelets. This monoclonal antibody was widely tested and verified by the authors via flow cytometry, immunohistochemistry, adhesion assays and immunoprecipitation (Massaguer et al., 2000, Tissue Antigens). As expected, treatment of platelets with calcium, thrombin and MA induced expression of CD62P which was detected by this antibody (Novus Biologicals website, Massaguer et al., 2003, Vet Immunol Immunopathol.).
DLK1 was tested by the manufacturer (Abcam). It detects recombinant DLK1 via Western blot, and shows the expected cell surface localization in U251 cells. Consistently, we found cell surface staining on glomerular ECs as predicted by our scRNA-seq data. Other studies have also found the highly specific expression of DLK1 in the expected cell populations (Eze et al. 2021 Nature Neuroscience).

Animals and other organisms

Policy information about studies involving animals; ARRIVE guidelines recommended for reporting animal research

Laboratory animals

Male C57BL/6N mice were put on a Western diet (Ssniff Spezialdiäten GmbH D12331) or control chow diet (Ssniff Spezialdiäten GmbH V1534) starting at 6 to 8 weeks of age. Mice were housed in an individual ventilated cage (IVT) system. Temperature was maintained at 22C (+/- 2C) and the humidity at 55% (+/- 10%). Water was provided ab liditum.

Wild animals

Study did not involve wild animals

Field-collected samples

Study did not involve field collected samples

Ethics oversight

All experiments were performed in accordance with the animal ethics laws of Saxony, Germany, and were approved by the state animal ethics committee (Landesdirektion Sachsen, Leipzig, Germany).

Note that full information on the approval of the study protocol must also be provided in the manuscript.

Flow Cytometry

Plots

Confirm that:

- [x] The axis labels state the marker and fluorochrome used (e.g. CD4-FITC).
- [x] The axis scales are clearly visible. Include numbers along axes only for bottom left plot of group (a 'group' is an analysis of identical markers).
- [x] All plots are contour plots with outliers or pseudocolor plots.
- [x] A numerical value for number of cells or percentage (with statistics) is provided.

Methodology

Sample preparation

Preparation of single cell suspensions
Brain. Brains were dissected and rinsed in ice-cold PBS. The olfactory bulb and cerebellum were removed. The brain was then dissociated with the Neural Dissociation Kit P (Miltenyi Biotec 130-092-628) as per the manufacturer's instructions via the gentleMACS™ Octo Dissociator system with heaters (MACS Technology, Miltenyi Biotec) using the "37C_NTDK_1" program. Cells were transferred via a 20 G syringe through a 70 µm cell strainer and into a 50 ml Falcon tube. Cells were collected by centrifugation (4°C, 300 g, for 5 min).

Lungs. The lungs were surgically removed, rinsed in ice-cold PBS, and transferred into a gentleMACS C tube (Miltenyi Biotec 130-096-334) containing tissue digestion buffer (TDB). TDB consisted of 1x penicillin/streptomycin (Thermo Fisher Scientific 15140122), 2x Antibiotic-Antimycotic, (Thermo Fisher Scientific 15240062), 1 mM sodium pyruvate (Thermo Fisher Scientific 1360070), 1x MEM Non-Essential Amino Acids Solution (Thermo Fisher Scientific 11140035), 0.13 WU Liberase TM (Merck 5401127001) and 160 U DNase I (Sigma-Aldrich D4527-10KU), made up in KnockOut™ DMEM (Thermo Fisher Scientific 10829018). Each sample was further dissociated with the gentleMACS™ Octo Dissociator system with heaters (MACS Technology, Miltenyi Biotec) using pre-programmed protocol "37C_m_LDK_1". The cell suspension was filtered through a 70 µm cell strainer and the cell strainer was rinsed once with 10 ml wash buffer (WB; containing 0.5% BSA (BSA Fraction V, Sigma-Aldrich 10735096001), 2 mM EDTA (Thermo Fisher Scientific 14190-094) in PBS). Cells were collected via centrifugation (4°C, 300 g, for 5 min).

Heart. The heart was surgically removed, rinsed in ice-cold PBS, cut into approximately 10 pieces and transferred into a gentleMACS C tube (Miltenyi Biotec 130-096-334) containing TDB supplemented with 0.13 WU Liberase TM (Merck 5401127001) and 80 U DNase I (Sigma-Aldrich D4527-10KU). Each sample was dissociated with the gentleMACS™ Octo Dissociator system with heaters (MACS Technology, Miltenyi Biotec) using the pre-programmed protocol "37C_NTDK_1". The cell suspension was transferred using a 20 G needle and filtered through a 70 µm cell strainer. The cell strainer was rinsed with 10 ml WB. Cells were collected by centrifugation and used for staining.

Kidneys. The kidneys were surgically removed and rinsed in ice-cold PBS. Kidneys were cut into small pieces using surgical scissors and transferred into a gentleMACS C tube (Miltenyi Biotec 130-096-334) containing TDB supplemented with 0.13 WU Liberase TM (Merck 5401127001) and 80 U DNase I (Sigma-Aldrich D4527-10KU). Each sample was dissociated with the gentleMACS™ Octo Dissociator system with heaters (MACS Technology, Miltenyi Biotec) using the pre-programmed protocol "37C_Multi_E". The cell suspension was filtered through a 70 µm cell strainer. The gentleMACS C tube and the cell strainer were subsequently rinsed in 10 ml WB. Cells were collected via centrifugation (4°C, 300 g, 5 min), washed once in 10 ml WB, and subsequently used for staining.

Liver. The liver was dissected, rinsed in ice-cold PBS, minced using scissors and transferred into a gentleMACS C tube (Miltenyi Biotec 130-096-334) containing TDB supplemented with 0.13 WU Liberase TM (Merck 5401127001) and 80 U DNase I (Sigma-Aldrich D4527-10KU). Each sample was dissociated with the gentleMACS™ Octo Dissociator system with heaters (MACS Technology, Miltenyi Biotec) using pre-programmed protocol "37C_m_LIDK_1". The cell suspension was filtered through a 70 µm cell strainer and further rinsed with 10 ml WB. Cells were collected by centrifugation (4°C, 300 g, for 5 min) and subsequently used for staining.

Adipose tissue. The visceral and subcutaneous AT were surgically removed, rinsed in ice-cold PBS, and transferred into a gentleMACS C tube (Miltenyi Biotec, 130-096-334) containing adipose digestion buffer (DMEM (Gibco 41966-029), 1% penicillin/streptomycin (Thermo Fisher Scientific 15140122), 0.2% Collagenase II (Gibco 17101-015)). Using surgical scissors, the adipose tissue was cut into small pieces. Samples were then dissociated with the gentleMACS™ Octo Dissociator system with heaters (MACS Technology, Miltenyi Biotec) using pre-programmed protocol "37C_mr_ATDK_1". The cell suspension was filtered through a 300 µm cell strainer. The gentleMACS C tube and the cell strainer were subsequently rinsed twice with 10 ml of WB. Cells were collected by centrifugation (4°C, 300 g, for 5 min). The adipose layer at the top was discarded, the cell pellet washed once in 10 ml WB, and the cells subsequently used for staining.

EC staining and isolation by FACS
For the 3-month timepoint (WD 3 months, chow 3 months), cell suspensions from the liver and kidneys were treated with RBC lysis buffer (0.154 M NH_4Cl, 0.01 M $KHCO_3$, 0.1 mM EDTA, 3 minutes, room temperature), washed once in WB, and subsequently used for staining. Myelin was removed from brain samples using myelin removal beads (Miltenyi Biotec 130-096-733) as per the manufacturer's instructions prior to staining. Similarly, epithelial cells were removed from lung suspensions using CD326 (EpCAM) microbeads (Miltenyi Biotec 130-105-958) according to the manufacturer's instructions.
For the 4-month (WD 4 months, chow 4 months, reversion 1 month) and 6-month (WD 6 months, chow 6 months, reversion 3 months) timepoints, cell suspensions from the liver, heart, kidneys, brain and lungs were treated with CD31 MicroBeads

	(Miltenyi Biotec 130-097-418) to enrich for ECs prior to staining. The CD31 enrichment was carried out according to the manufacturer's instructions.<br>All samples were stained with the CD45-PE (BD Pharmigen 533081, 1:400) and CD31-APC (eBioscience 17-0311-85, 1:250) antibodies, diluted in FACS buffer (2% FCS in PBS). Staining was done on ice in a total volume of 200 µl. Cells were washed in 14 ml of FACS buffer, collected by centrifugation (4°C, 300 g, for 5 min), resuspended in FACS buffer containing 1 µg / ml propidium iodide and passed through a 100 µm cell strainer into a FACS tube.
Instrument	FACS Melody (BD Biosciences).<br>FACS Aria (BD Biosciences)
Software	Flowjo v10.6.2
Cell population abundance	FACS sorted cells were used for single cell RNA-seq analysis.<br>More than 85% of the cells in the CD31+ CD45-low gate were endothelial cells according to our single cell analysis. Endothelial cells were identified based on well established markers (Cdh5, Pecam1, Flt1). Any contaminating mural cells, fibroblasts and immune cells were identified based on the expression of known markers and removed from the analyses.
Gating strategy	Debris was removed using the FSC-A versus SSC-A gate, with the smallest events being removed. Single cells were selected based on forward and side scatter (FSC-H versus FSC-W; SSC-H versus SSC-W; FSC-A versus FSC-H). Dead cells were removed using propidium iodide. ECs were gated based on CD31pos and CD45low expression.

☒ Tick this box to confirm that a figure exemplifying the gating strategy is provided in the Supplementary Information.
